# First Demonstration of Antigen Induced Cytokine Expression by CD4-1^+^ Lymphocytes in a Poikilotherm: Studies in Zebrafish (*Danio rerio*)

**DOI:** 10.1371/journal.pone.0126378

**Published:** 2015-06-17

**Authors:** Sohye Yoon, Suman Mitra, Cathy Wyse, Ayham Alnabulsi, Jun Zou, Eveline M. Weerdenburg, Astrid M. van der Sar, Difei Wang, Christopher J. Secombes, Steve Bird

**Affiliations:** 1 Scottish Fish Immunology Research Centre, School of Biological Sciences, University of Aberdeen, Aberdeen, United Kingdom; 2 Vertebrate Antibodies Ltd, Zoology Building, University of Aberdeen, Aberdeen, United Kingdom; 3 VU University, Medical Center, Department of Medical Microbiology & Infection Control, 1081 BT Amsterdam, Amsterdam, The Netherlands; 4 Department of Biochemistry and Molecular & Cellular Biology, Georgetown University Medical Center, Washington, United States of America; Friedrich-Loeffler. Institut, GERMANY

## Abstract

Adaptive immunity in homeotherms depends greatly on CD4+ Th cells which release cytokines in response to specific antigen stimulation. Whilst bony fish and poikilothermic tetrapods possess cells that express TcR and CD4-related genes (that exist in two forms in teleost fish; termed CD4-1 and CD4-2), to date there is no unequivocal demonstration that cells equivalent to Th exist. Thus, in this study we determined whether CD4-1^+^ lymphocytes can express cytokines typical of Th cells following antigen specific stimulation, using the zebrafish (*Danio rerio*). Initially, we analyzed the CD4 locus in zebrafish and found three CD4 homologues, a CD4-1 molecule and two CD4-2 molecules. The zfCD4-1 and zfCD4-2 transcripts were detected in immune organs and were most highly expressed in lymphocytes. A polyclonal antibody to zfCD4-1 was developed and used with an antibody to ZAP70 and revealed double positive cells by immunohistochemistry, and in the *Mycobacterium marinum* disease model CD4-1^+^ cells were apparent surrounding the granulomas typical of the infection. Next a prime-boost experiment, using human gamma globulin as antigen, was performed and revealed for the first time in fish that zfCD4-1^+^ lymphocytes increase the expression of cytokines and master transcription factors relevant to Th1/Th2-type responses as a consequence of boosting with specific antigen.

## Introduction

T helper (Th) cells play a key role in adaptive immunity by secreting cytokines that initiate and activate downstream effector mechanisms. The CD4 molecule is a transmembrane protein expressed on the surface of Th cells where it functions as a co-receptor with the TcR by binding to MHC class II molecules on the surface of dendritic cells, that present antigens [1, 2]. CD4^+^ T cells in mammals are divided into a number of effector subpopulations, that elicit appropriate immune responses to different pathogen/antigen types by releasing different repertoires of cytokines [3, 4]. Th1 and Th2 cells were discovered initially [5] but other recently characterized effector Th subpopulations include Th9 [6], Th17 [7], Th22 [8] and T follicular helper (Tfh) [9] cells. Naïve CD4^+^ cells differentiate into the different subpopulations after antigen stimulation, dependent upon the cytokine milieu that drives expression of master transcription factors associated with each Th subset [3]. Whilst these recent advances tell us a lot about CD4^+^ Th cell plasticity and role in immunity and disease states in mammals, we know very little about the Th cell populations that may exist in other vertebrate groups.

It is clear that T and B cells exist in all jawed vertebrates, with extensive analysis of the T and B cell receptor repertoires in several non-mammalian groups, including Osteichthyes [10, 11]. The identification of CD4/MHC II and CD8/MHC I molecules in all euteleostomi (tetrapods and bony fish), although there may have been secondary loss of some of these molecules in certain fish species, as in cod [12], hints that T cell subpopulations will be a universal feature of the adaptive immune system of these organisms. Indeed, recent studies in bony fish have confirmed the role of CD8^+^ T cells in specific cytotoxicity [13–15], and so at least this arm of the T cell system appears to have been present in the early Osteichthyan ancestor, and has remained conserved in bony fish and tetrapods. Far less is known about Th evolution and Th cell subpopulations in early vertebrates, and a recent study questions whether more than Th1 cells will exist in cartilaginous fish [16].

In teleost fish a CD4-like (CD4-1) molecule has been isolated from many species, including fugu [17], rainbow trout [18], carp [19], catfish [20], sea bass [21], Atlantic halibut [22], Atlantic salmon [23], and Japanese flounder [24]. It contains the typical four immunoglobulin domains and an lck binding site. In addition, a second CD4 related (CD4-2 or CD4-rel) gene has been identified in several teleost species, including trout [18, 25], catfish [20], salmon [23], tetraodon [26] and Japanese flounder [24] that contains fewer (2–3) Ig domains. Thus, whilst molecules with homology to CD4 are known to exist in fish, the role(s) of CD4-1^+^ or CD4-2^+^ T cells within the adaptive immune response is still largely unclear. Such studies have been hampered by the lack of appropriate tools to identify T cells but recently antibodies to ginbuna crucian carp CD4-1 and fugu CD4-1 have been produced and employed to study CD4-1^+^ cells in these species [26–28]. In ginbuna crucian carp the CD4-1^+^ cells were negative for surface IgM but positive for TcRβ transcripts. Whilst very few CD4-1^+^/CD8^+^ cells were detected in peripheral leucocytes, they represented 16% of lymphocyte gated cells in the thymus. In addition, isolated ginbuna CD4-1^+^ cells were found to proliferate *in vitro* in response to allogeneic or specific antigen (ovalbumin—OVA) stimulation. In fugu CD4-1^+^ cells were shown to express transcripts for both CD4-1 and CD4-2 but were negative for CD8α. Stimulation of the isolated cells with PAMPs or a T cell mitogen (ConA) *in vitro* elicited expression of several cytokines of adaptive immunity. More recently, two studies in ginbuna crucian carp show that CD4-1^+^ cells play a role in protection against bacterial and viral pathogens, using adoptive transfer of MACS sorted cells from sensitized syngeneic fish [29, 30]. In the case of CD4-2^+^ cells, one study has shown that when co-expressed with CD25 the CD4-2^+^ cells have an apparent Treg phenotype [26]. However, to date there has been no demonstration of antigen-specificity in the cytokine response of CD4-1^+^ or CD4-2^+^ cells in fish.

To address this issue, in the present study we have examined the cytokine expression in zebrafish CD4-1^+^ lymphocytes following antigen restimulation. To be able to perform these experiments, a detailed analysis of the CD4 locus in zebrafish was initially undertaken, where three genes with relatedness to CD4 are clustered; CD4-1, CD4-2.1 and CD4-2.2. The CD4-2.2 molecule has no apparent intracellular region and thus whether it will be functional remains to be seen. Whilst both CD4-1 and CD4-2 could be important in terms of their ability to trigger responses in Th, in this study we focused on CD4-1. A specific antiserum to zebrafish CD4-1 was generated and several parameters of the CD4-1^+^ cells investigated, including whether they co-expressed the T cell marker ZAP70, if they could be detected in the cellular response to *Mycobacterium marinum* infection, and their expression of relevant genes in sorted cells. Finally an *in vivo* prime-boost model was established using human gamma globulins (HGG) as antigen, known to be an excellent immunogen in cyprinids [31–33], and the cytokine profile examined in lymphocyte gated CD4-1^+^ cells following specific antigen restimulation *in vivo*, in comparison to the response in cells from fish given HGG for the first time or an unrelated antigen (OVA). Our data clearly demonstrate that fish CD4-1^+^ cells, sorted from the lymphocyte gate and with a lymphocyte morphology, can be induced to express cytokines of adaptive immunity following antigen restimulation.

## Materials and Methods

### Animals

Adult zebrafish (zf), *Danio rerio* (Wik strain), were obtained from Queen’s Medical Research Institute Zebrafish Facility, University of Edinburgh, and maintained in a recirculation system (Tecniplast) in the University of Aberdeen zebrafish aquarium at 28°C. Fish were fed with tropical fish flake twice daily and frozen brine shrimp and blood worm twice a week. Fish were given at least 1 week for acclimatization prior to treatment. All procedures were carried out under the UK Animals (Scientific Procedures) Act 1986 and Home office code of Practice guidance, under Home Office project license PPL 60/4013. The zebrafish used in this study were anaesthetized in MS-222 prior to procedures and every effort was made to minimize suffering during the experiments performed.

### RNA extraction, cDNA synthesis, RACE PCR and molecular cloning

The zfCD4-1 sequence was initially found on zebrafish chromosome 16 by searching the Ensemble zebrafish genome assembly version 9 (zv9, www.ensemble.org/Danio_rerio/) using BLAST [34] with amino acid sequences of known fish CD4-1 molecules. A large region around this sequence was subsequently analyzed for possible coding regions using Genscan [35] which identified the potential zfCD4-2 genes. This approach along with sequence information identified from available EST’s (CF999671, DR725676, CN015696, EB981233, EB925541) allowed the construction of partial coding regions for zfCD4-1 and zfCD4-2 cDNA, which were used to design primers (S1 Table) to allow the amplification of the full length cDNA using rapid amplification of cDNA ends (RACE). Total RNA for 5’ and 3’ RACE was isolated from spleen and kidney tissues, under RNase free conditions. Following addition of TRI reagent (Sigma) to the samples, tissues were stored at -80°C until use. Next, chloroform (Sigma) was added, the aqueous phase was transferred to a fresh tube and an equal volume of isopropanol was added to precipitate the RNA at -80°C for at least 1 h. After centrifugation at 4°C, the supernatant was discarded and 70% ethanol was then added to wash the RNA pellet. Lastly, RNA was re-suspended in RNase/DNase free water and the concentration measured. Either normal cDNA was synthesized using both Oligo (dT)_18_ and a random primer (S1 Table) or cDNA for 3’RACE was synthesized using the adapter dT primer (S1 Table) from total RNA, using a RevertAid First Strand cDNA Synthesis Kit (Fermantas) according to the manufacturer’s instructions. To amplify the 3’ end of zfCD4-1, the first round PCR used CD4-1raceF1 and the adapter primer (S1 Table) and the product of this was used in a semi-nested PCR with CD4-1raceF2 and the adapter primer (S1 Table). cDNA for 5’RACE was synthesized by adding RNase H (Promega) to 3’ RACE cDNA or normal cDNA and incubated for 20 min at 37°C. The cDNA samples were purified with a QIAquick PCR purification kit (Qiagen) according to the manufacturer’s instructions. Nuclease-free water was added to re-suspend the purified cDNA followed by recombinant TdT (rTdT) 5x buffer (Promega) and 2mM dCTP. After incubation for 3 min at 94°C to denature the cDNA, rTdT (Promega) was added and the mix incubated for 60 min at 37°C. The reaction was stopped by heating at 72°C for 10 min, when the synthesized cDNA with a 5’poly(dC) tail was ready for use. To amplify the 5’ end of zfCD4-1, the first round PCR was performed using the CD4-1raceR1 primer and oligo dG (S1 Table), and the product of this was used in a semi-nested PCR with CD4-1raceR2 and oligo dG (S1 Table). The 3’ and 5’ ends of the CD4-2.1 and CD4-2.2 genes were amplified in the same way. The primers used in the experiments are listed in S1 Table. Full length transcripts of the three genes obtained from the RACE PCR were then confirmed by full length PCRs.

Obtained PCR products were ligated into pGEM-T easy vector (Promega) and transformed into competent *Escherichia coli* cells (ActifMotif). Positive cells were screened and plasmid DNA from at least three independent clones was purified using a QIAprep Plasmid DNA Miniprep kit (Qiagen) according to the manufacturer’s instructions, and sequenced by Eurofins MWG Operon (Germany).

### Bioinformatics analysis

Sequences were analyzed for similarity to other known molecules using FASTA [36] and the Basic Local Alignment Search Tool (BLAST) suite of programs [34]. The predicted amino acid (aa) sequences obtained from the Expert Protein Analysis System (ExPASy) proteomics server of the Swiss Institute of Bioinformatics (SIB) [37] were analyzed using Simple Modular Architecture Research Tool (SMART) and the TMpred programme to confirm putative protein structure. Multiple sequence alignments were generated by ClustalW (Version 1.60) and phylogenetic relationships were established using the neighbour-joining method and complete deletion treatment for gap data with full length protein sequences of the known CD4-1 and CD4-2 molecules using MEGA5 software [38]. Transcription factor binding sites in the CD4-1 promoter region were predicted using Genomatix software (Version 2.7). The zfCD4-1, zfCD4-2.1 and zfCD4-2.2 gene organization were elucidated using the full length cDNA obtained by RACE PCR and the zebrafish genome. The cDNA was aligned with the appropriate region within zebrafish chromosome 16 using Spidey [39]. BLAST [34], Genscan [35] and FASTA [36] were used to predict genes within the CD4 locus of the zebrafish, stickleback and tetraodon genome and to examine the synteny that exists between them and the CD4 locus within the human genome. Protein sequence similarity and identity were determined using the programme MatGAT, with the BLOSUM62 alignment matrix and default settings for gap insertion [40]. The Modeller package [41] was used to build the homology models of zfCD4-1, zfCD4-2.1 and zfCD4-2.2 proteins bound to the corresponding zfMHC class II (MHCII) dimer, to assess the potential for these molecules to act as coreceptors on T cells. We first aligned MHCII α and β chain sequences from zebrafish, mouse and human using ClustalW [42] in the BioEdit program. Based on the MHCII and CD4 alignment results (S1 and S2 Figs), the homology models were built for zebrafish CD4-1, CD4-2.1 and CD4-2.2 and the MHCII complex, using the X-ray structure of binary complex of human CD4 N-terminal two-domain fragment and mouse MHCII molecule [PDB: 1JL4] [43] as the template structure.

### Expression analysis of CD4-1 and CD4-2 genes in zebrafish

#### Expression distribution of zfCD4-1 and zfCD4-2 genes in different tissues

To determine the distribution pattern of the zfCD4-1, zfCD4-2.1 and zfCD4-2.2 transcripts, blood and tissues from freshly killed adult zebrafish were collected, with the latter including kidney, spleen, gill, liver, intestine and muscle. After extraction of total RNA using RNA TRI reagent (Sigma) and synthesis of cDNA using the RevertAid First Strand cDNA Synthesis Kit (Fermantas), the samples were analyzed by real time PCR (QRT-PCR) using the primer sets for zfCD4-1, zfCD4-2.1 and zfCD4-2.2, shown in S2 Table.

#### Expression of zfCD4-1 and zfCD4-2 genes in different populations of leukocytes sorted by flow cytometry

For leukocyte expression analysis, kidney and spleen were used. The tissues were pooled from 4–5 fish, and pushed through a 100μm pore size medicon mesh (BD Science) in PBS with 2% FCS (Sigma), and then through a 70μm pore size filter to exclude aggregated cells which may affect flow cytometry analysis. The cells were centrifuged at 400g for 5 min at 4°C, and the pellet was re-suspended in PBS with 2% FCS. Approximately 1×10^6^ cells were used for cell sorting using a FACS Vantage SE (Becton Dikinson) on the basis of size and shape (complexity) in the forward scatter and side scatter plots. Each gate was checked for purity post cell sorting. The cell populations isolated were analyzed by QRT-PCR, following RNA extraction and cDNA synthesis as above, using the primer sets for zfCD4-1, zfCD4-2.1 and zfCD4-2.2 (S2 Table).

### Real time PCR

Real time PCR was performed using GoTaq qPCR Master Mix (Promega) on a light Cycler 480 real time PCR machine (Roche). The PCR program used for amplification was as follows: 1 cycle of 94°C for 10 min, 40 cycles of 94°C for 20 s, annealing at 60°C for 20 s and extension at 72°C for 20 s, followed by 1 cycle of 72°C for 10 min. A melting curve for each PCR reaction was established between 72°C and 94°C to ensure only a single product was amplified. For quantification of target genes, analysis of expression was performed by normalizing the cycle threshold (CT) value of target genes to that of the internal control Elongation Factor (EF)-1α.

### Detection of zfCD4-1^+^ cells

#### Antibody production and validation of polyclonal anti zfCD4-1 serum

Two peptide sequences corresponding to positions 31–44 and 151–164 of the zfCD4-1 polypeptide described in this work were predicted to be hydrophilic on the external side of the CD4-1 protein, and were used for polyclonal antiserum generation by Biogenes in Germany. These two peptide sequences were unique relative to the sequence of the zfCD4-2 genes. The individual peptides were synthesized and then conjugated to keyhole limpet hemocyanin (KLH) using glutaraldehyde, and a mixture of the two peptide conjugates were used for immunization of two New Zealand white rabbits. Pre-immune serum from the same rabbits was collected prior to immunization commencing. The antibody reactivity to each peptide was determined by testing serum from immunized animals by ELISA. The serum with the highest antibody titre was used further, as outlined below.

#### zfCD4-1 expression in transfected mammalian cells

The full length ORF of zfCD4-1, excluding the start/stop codons, was amplified and ligated into a modified pEF6/V5-His vector (Invitrogen) as described previously [44]. This construct translated the full-length zfCD4-1 followed by a C-terminal V5 polyhistidine tag (GKPIPNPLLGLDSTRTGHHHHHH). The plasmid encoding zfCD4-1 was sequence confirmed (Eurofins MWG Operon, Germany) and then used for transfection of Chinese Hamster Ovary (CHO) cells. The transfection was performed using PEI (Polysciences) following the manufacturer’s protocol. After culture for 24 h at 37°C and then for 48 h at 32°C, the cells were centrifuged and used in Western blotting and FACS analysis to demonstrate the expression of zfCD4-1. For Western blotting, the pellet was re-suspended in lysis buffer (150mM NaCl, 150mM Tris-HCl, 5mM EDTA, 9M Urea, 2% SDS and 0.2% Triton 100) and stored at -80°C for freezing and thawing to lyse the cells. 2mM phenylmethanesulfonylfluoride (Sigma) was added to the cell lysate before carrying out Western blot analysis. Un-transfected CHO cells were used as a control. For FACS analysis, the pellet was re-suspended in PBS containing 2% FCS and then used as outlined below.

### Western blotting

Cell lysates from zf kidney and spleen and transfected CHO cells were added to NuPAGE LDS 4x sample buffer (Invitrogen) and boiled at 95°C for 10 min. The samples were resolved on a 4–12% SDS polyacrylamide gel (Invitrogen) and transferred onto PVDF membranes (Millipore). The membranes were blocked with 5% non-fat dry milk in TBST buffer (20mM Tris-HCl, pH 7.4, 150mM NaCl, 0.1% Tween 20) and probed with the zfCD4-1 polyclonal antibody diluted 1:200 as the primary antibody and anti-rabbit IgG-peroxidase antibody (Sigma) as the secondary antibody diluted 1:2000. A SuperSignal West Pico Trial Kit (Thermo Scientific) was used as the enhanced chemiluminescent HRP substrate to detect the proteins.

### Immunofluorescence

Zebrafish kidney leukocytes were prepared as for flow cytometry (see above) and white blood cells were centrifuged onto microscope slides using a Shandon Cytospin3 (Thermo Electron). The slides were incubated with the zfCD4-1 antibody diluted 1:200 followed by FITC conjugated anti-rabbit IgG (BD Science) diluted 1:500, and then examined under a fluorescence microscope (Zeiss). To verify the surface staining of the cells, in addition some cells were examined using a confocal fluorescence microscope (LSM700, Zeiss Imager M2 Upright Microscope).

### Double immunofluorescence analysis of frozen tissue sections

A rabbit monoclonal anti-human ZAP70 (BD 99F2, BD science) was purchased and used for double staining with the polyclonal anti-zfCD4-1 serum in immunohistochemistry. This antibody has been shown previously to react with carp thymocytes but not B cells, macrophages or granulocytes [45], and we confirmed that it detects a 70 kDa protein in Western blotting with zebrafish leukocyte lysates (S3 Fig). Zebrafish were killed and fixed overnight in 4% paraformaldehyde (pH 7.4), then transferred into cryoprotectant sucrose (30%) in 0.1M phosphate buffer (pH 7.4) for 24 h. The whole fish body was then placed onto dry ice for quick freezing and sectioned at 30μm on a freezing microtome (Leica). The slides were kept in 0.1M citrate buffer (pH 6.0) and autoclaved for antigen retrieval. The rabbit anti-zfCD4-1 serum (diluted 1:5000) and a donkey anti-rabbit IgG Alexa Flour 594 secondary antibody (diluted 1:200) (Molecular Probes, Invitrogen) were used for detection of CD4-1^+^ cells. The mouse anti-ZAP70 serum was diluted 1:50 and a goat anti-mouse IgG Alexa Flour 488 secondary antibody was diluted 1:200 (Molecular Probes, Invitrogen) for detection of ZAP70^+^ cells. Tissue sections were incubated for 1 h at room temperature with the primary and secondary antibodies, in 0.1M Tris buffer containing 2% normal horse serum. A nuclear counter stain was performed by incubation with a ToPro3 dye solution (1:1000) (Invitrogen). The slides were then washed x3 in 0.1M Tris buffer for 10 min, coverslipped using an aqueous mounting medium (Glycerogel, Dako), and stored at 4°C in the dark. Immunopositivity was detected using a Zeiss 710LSM confocal laser-scanning microscope (Zeiss, Jena), in x-y plane, and in z-stack images, when co-localization of immunopositivity was suspected. The pinhole aperture was standardized at 1 unit for all channels. Co-localization of signals was accepted when signals were detected in separate channels without physical signal separation in optical slices of ≤1 μm using 40x (Plan-Neofluar 40x/1.30) or 63x (Plan-Apochromat 63x/1.40) lenses. Specific immunopositivity was absent in sections processed in the absence of primary antibody or using primary antisera pre-adsorbed with the immunizing peptide (in the case of CD4-1). Images were processed using ZEN software (Zeiss).

### Immunohistochemical analysis of sections of M. marinum infected zebrafish

In a further experiment to look at the involvement of CD4-1^+^ cells during disease states, the granulomas that develop in zebrafish in response to *M*. *marinum* infection were examined, for the presence of CD4-1^+^ cells in this cellular reaction. One-year old male zebrafish were infected for four weeks with *M*. *marinum espG*_*5*_::Tn, or eight weeks with the wild-type strain [46], by intraperitoneal injection with 2 x 10^4^ cfu/fish or 10 μl phosphate buffered saline (PBS) as control, and fixed in 4% paraformaldehyde after terminal anaesthesia. Fixed animals were embedded in paraffin and cut in coronal serial sections from ventral to dorsal. Tissue sections were deparaffinized in xylene and rehydrated up to 70% alcohol. Endogenous peroxidase was inactivated by incubation in 0.3% H_2_O_2_ in methanol for 30 min. After 10 min of pre-incubation in 1% BSA, sections were incubated with the polyclonal anti zfCD4-1 antibody diluted 1:1000 in 0.1% BSA for 60 min in a humidified chamber. Slides were washed with PBS and incubated with a horseradish peroxidise conjugated goat anti-rabbit antibody (Rockwell) in 0.1% BSA for 30 min. After washing with PBS, slides were stained with 0.05% DAB/0.02% H_2_O_2_, counterstained with haematoxylin and eosin and dehydrated with xylene. All incubations were performed at room temperature. A Zeiss Axioskop light microscope equipped with a Leica DC500 camera was used for imaging. ImageJ software was used to adjust the brightness and contrast of images.

### FACS analysis

For the detection of zfCD4-1 expression in zfCD4-1 transfected CHO cells, cells from the above section ‘zfCD4 expression in transfected mammalian cells’ were incubated with the anti-zfCD4-1 serum, followed by FITC-conjugated anti-rabbit IgG. Pre-immune serum from the same rabbit was used at the same dilution as the CD4-1 serum as a negative control, to test for non-specific binding.

For the detection of zfCD4-1 expressing cells in zebrafish, kidney and spleen cells were prepared as outlined above and leukocytes isolated using 51% Percoll (Sigma) gradients. The cells were blocked by incubation in PBS + 2% FCS for 30 min on ice and then incubated with the anti-zfCD4-1 serum (diluted 1:200), washed and further incubated with the secondary antibody, FITC-conjugated anti-rabbit IgG (BD Science) diluted 1:500. FACS analysis was then undertaken with an Accuri C6 Flow Cytometer (BD Science). In this case the negative controls consisted of pre-immune rabbit serum (diluted 1:200) or no primary antisera.

Lastly, zfCD4-1^+^ and CD4-1^-^ cells were sorted as described below, and analyzed for the transcript level of different cell surface markers by real time PCR (using primers in S2 Table), to determine whether differences in these two populations were apparent.

### Immunization study

This experiment was undertaken to examine whether evidence could be found for antigen-specific induction of cytokines of adaptive immunity in the zfCD4-1^+^ cell population. Two experiments were undertaken. The first to show that the experimental model worked in terms of inducing detectable transcript levels of relevant cytokines. The second was a repeat that included FACS sorting of CD4-1^+^ cells from the lymphoid population only, with subsequent analysis of cytokine expression and a variety of cell surface markers.

#### 1^st^ experiment

Two groups of 36 fish were injected i.p. with either human gamma globulins (HGG, Sigma) dissolved in PBS and then emulsified in Freund’s complete adjuvant (FCA, Sigma), or PBS alone but also emulsified in FCA. PBS or 4 mg/ml HGG were emulsified in an equal volume of FCA and 5μl injected per 0.5g bw (i.e. 10μg/ 0.5g fish). The fish were left for 8 weeks and then boosted. Each group was divided into two subgroups of 18 fish and boosted with either HGG or ovalbumin (OVA, Sigma) (10μg/0.5g fish) emulsified in an equal volume of Freund’s incomplete adjuvant (FIA). In this way we could examine cytokine gene expression following boosting with a specific (HGG) or non-specific (OVA) antigen in comparison to fish given only a primary immunization to either antigen. In addition, a further group was injected with saline at this time. For analysis of cytokine expression, 6 fish per group were killed at day 1, 3 and 7 post boosting, and the kidney isolated for individual fish RNA extraction and cDNA synthesis as above. QRT-PCR analysis was carried out, using primer sets (S2 Table) for IFN-γ, IL-4/13A and IL-4/13B expression [47], as potential markers of Th1 and Th2 responses respectively.

#### 2^nd^ experiment

The experimental design was the same as for the 1^st^ experiment with the exception that the number of fish was increased to allow sufficient cells to be obtained for QRT-PCR analysis of sorted CD4+ cells from the gated lymphoid population. In this case 20 fish were killed per subgroup per sampling time post boosting. The kidney and spleen of 5 fish were pooled, to obtain a sufficient quantity of leukocytes for FACS analysis and cell sorting, giving four biological replicates per group per time. The cells were incubated with the anti-zfCD4-1 serum followed by an allophycocyanin (APC, BD Science) fluorochrome-conjugated secondary antibody diluted 1:500. ZfCD4-1^+^ lymphocytes were then sorted from the lymphocyte gate using the relative fluorescence level in comparison to negative control cells which were only stained with secondary antibody, with a BD Influx cell sorter (BD Science). The sorting purity was ≥98% and the cells were visualized by hematoxylin/eosin staining on the sorted cells from the lymphocyte gate. The sorted zfCD4-1^+^ lymphocytes underwent RNA extraction and cDNA synthesis as above. QRT-PCR analysis was carried out using primer sets (S2 Table) for expression of cytokine genes, transcription factors and selected cell surface markers (the latter in comparison to unsorted cells). The genes studied included potential markers of B-cells (IgM and MHCIIβ), T-cells (zfCD4-1, zfCD4-2.1, zfCD4-2.2 and TcRα), Th17 cells (IL-22 and IL-17A/F, the latter having homology to both IL-17A and IL-17F [48]), as well as the master transcription factors Tbet and GATA3 for Th1 and Th2 respectively [49].

### Statistical analysis

A two tailed paired Student’s T-test was used to determine whether the means were significantly different between control and treated groups and to check for significant differences in gene expression between zf leukocytes sorted into lymphocytes, haemopoietic precursors or monocytes/granulocytes. Values were considered to be significant when P<0.05 using Prism software. For the immunization study, 6 individual fish were used for gene expression analysis in the first experiment and 4 independent biological replicates (of cell pools from 5 individuals) were used for analysis in the second experiment. All the cDNA samples were run in triplicate for PCR expression analysis.

## Results

### Sequence analysis and characterization of zfCD4-1/CD4-2 molecules

**Fig 1:**
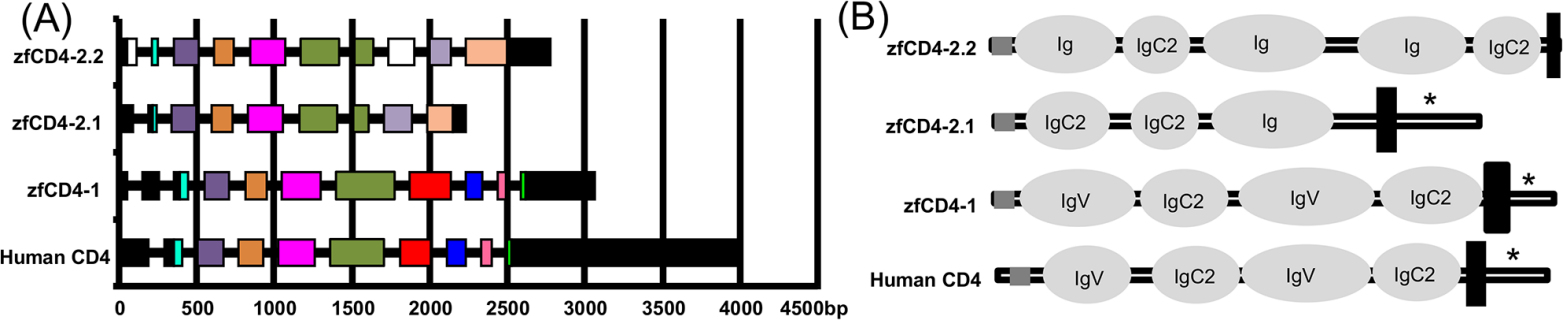
Schematic representation of human CD4 and zebrafish CD4-1/CD4-2 gene organization and structure. (A) Gene organization of zebrafish CD4-1, CD4-2.1, CD4-2.2 and human CD4. Equivalent exons are in the same colour. (B) Structural analysis of the zfCD4 related proteins compared to human CD4. The predicted Ig domains (pale grey), signal peptide (grey) and transmembrane region (black) are shown. The lck binding motif of zfCD4-1, zfCD4-2.1 and human CD4 is indicated with an asterisk.

Full length cDNA sequences for three zfCD4-related genes were obtained using 3’ and 5’ RACE PCR with primers designed to sequences identified from searching the zebrafish genome and EST database. The sequences were designated as zfCD4-1 (Accession no. HE983359), zfCD4-2.1 (Accession no. HE983358) and zfCD4-2.2 (Accession no. HE983357). BLAST analysis revealed they were located on zebrafish chromosome 16 (zv9, www.ensemble.org/Danio_rerio/). The zfCD4-1 gene consists of a single open reading frame of 1419 bp encoding a 474 aa protein with a predicted molecular mass of 53 kDa. The zfCD4-2.1 and zfCD4-2.2 genes encode proteins of 411 aa (46 kDa) and 515 aa (56.5kDa), respectively. The zfCD4-1 gene has 11 exons, differing from the human CD4 gene which consists of 10 exons (Fig 1 (A)), with the extra intron in the 5’ untranslated region. The zfCD4-2.1 and 2.2 genes have 9 exons and 10 exons, respectively. Protein analysis predicted that the structure of zfCD4-1 matches that of human CD4, in containing a signal peptide and 4 Ig domains in the extracellular region, followed by a transmembrane domain and intracellular region (Fig 1 (B)). The zfCD4-2.1 shows a similar domain organization but has only 3 predicted Ig domains. In contrast, the zfCD4-2.2 protein was predicted to contain 5 extracellular Ig domains and to lack an intracellular region.

Multiple sequence alignment was performed to examine conserved regions in the zfCD4-related proteins compared with CD4/CD4-1/CD4-2 and Lymphocyte-Activation Gene 3 (LAG3) proteins in human and other fish species (S4 Fig). LAG3, or CD223, is also a CD4 related molecule that exists in fish as well as mammals. A WTC motif in domain 2 was seen in all CD4 aa sequences examined, and most of the teleost CD4-1 proteins had a potential N-glycosylation site (NXS/T). A CXC (p56lck) motif was also present in the C-terminal intracellular region of all the CD4-1/CD4-2 molecules but not in LAG3, with the exception of zfCD4-2.2 which has no intracellular region.

**Fig 2:**
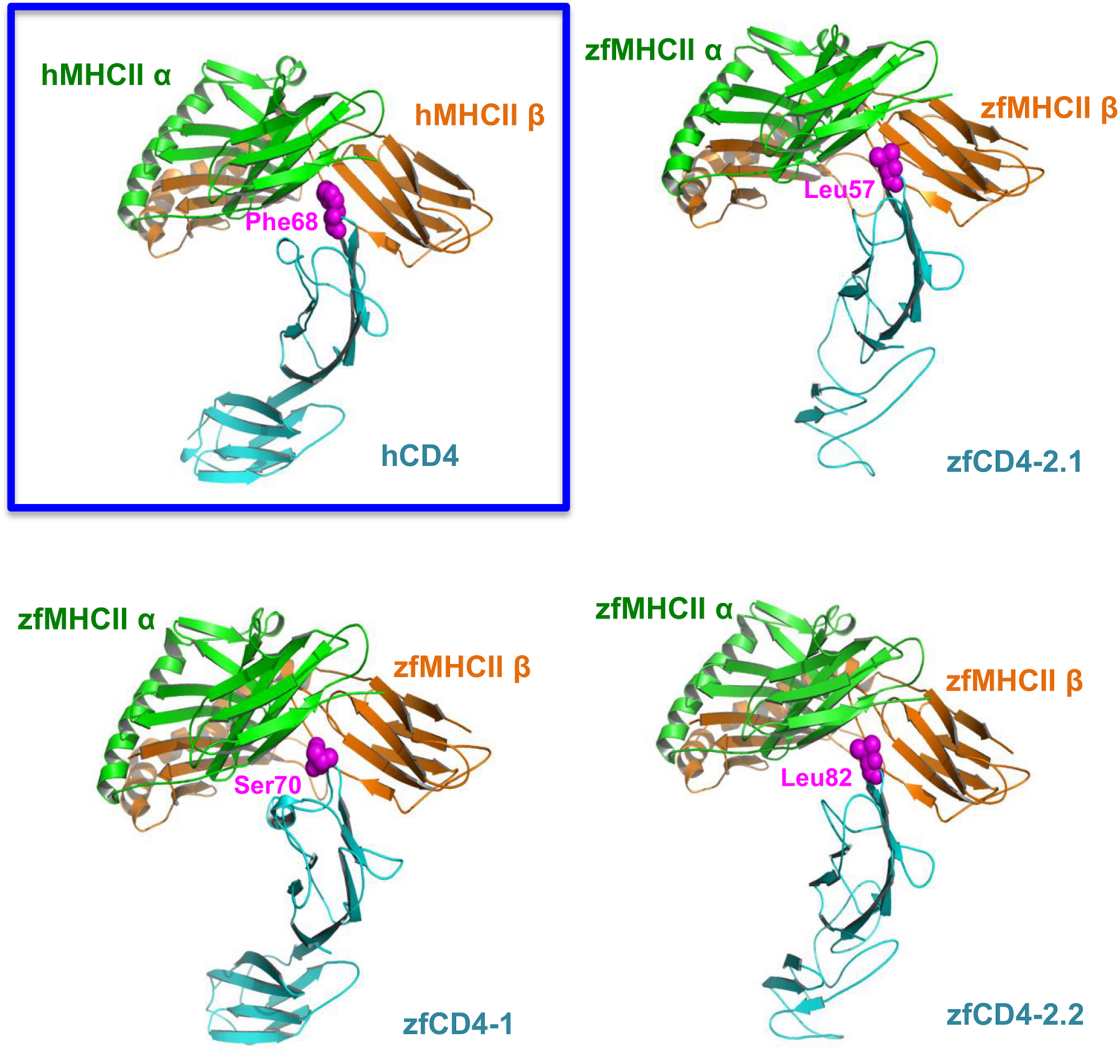
Structural comparison of MHCII-CD4 complexes of human (top, left) and zebrafish. The residues that interact with the hydrophobic area formed by MHCII α and β chains are shown in magenta balls.

An important function of mammalian CD4 is to interact with MHC class II molecules, and thus we constructed 3D models for the predicted zfCD4-1 and CD4-2 proteins along with the zfMHC II molecule. Both MHCII chains (the α2 and β2 domains) in zebrafish, mouse and human MHC II show considerable sequence similarities (S1 and S2 Figs), and it is reasonable to postulate that these proteins may fold into similar structures. In addition, the residues (marked as red ovals) at the interface between MHCII α2 and β2 are very conserved across the three species. Hence we expected the structure of the MHCII α2 and β2 complex to be very similar for human, mouse and zebrafish, and the models confirmed these expectations (Fig 2). The interactions between the MHCII dimer and CD4 are quite similar in the three species. The homology models of zebrafish CD4-2.1 and CD4-2.2 with their MHCII complex showed that Leu57 and Leu82, like Phe43 in PDB 1JL4 (Phe68 in human CD4 = Phe43 in Wang et al., [43] and Yin et al., [50], are close to the hydrophobic area formed by Ile108/Val118, Tyr109/Phe119 and Trp196/Trp205 (zebrafish/mouse) from MHCII α2 and Met167/Ile174 and His177/Leu184 from MHCII β2. However, such a residue is missing in the zfCD4-1 sequence. In the case of zfCD4-1 the model shows that the domain core consists of hydrophobic amino acids like Ile23, Ala25, Val31, Leu33, Trp54, Ile88, Val91, Trp99, Tyr110 and Tyr114, and it is possible this domain will still form an Ig domain even though the typical cysteine pair (or S-S bridge) is missing. For zfCD4-1 it is Ser70 that is modeled to interact with MHCII at the hydrophobic area. Whether such differences may influence the potential activation of T cells expressing these molecules will require further experimental validation but the models do suggests both CD4-1 and CD4-2 are functional in terms of their ability to act as co-receptors for MHCII. (Note: we have used the residue numbers in the canonical sequences in UniProt, http://www.uniprot.org. In the X-ray structure of mouse MHCII and human CD4 complex, PDB 1JL4, residue numbers are all shifted in Chain A, B and D. The corresponding UniProt IDs for MHCII sequences used here are P01903 and P04229 for human, P01910 and P06343 for mouse).

**Fig 3:**
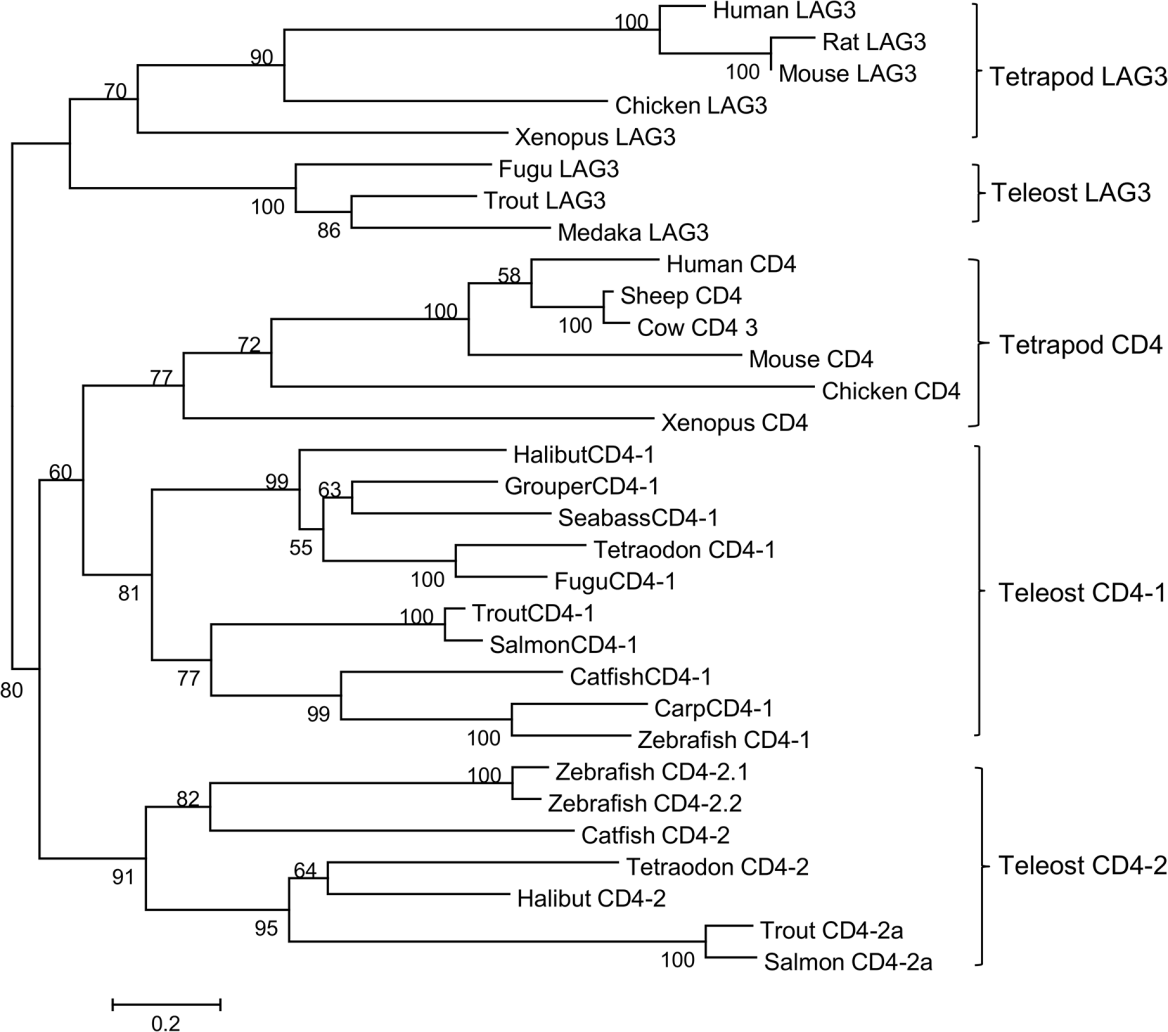
Phylogenetic tree showing the relationship between vertebrate CD4, CD4-1, CD4-2 and LAG3 proteins. The amino acid sequences of the genes were aligned using clustal W and the tree constructed by the NJ method and supported with 1000 bootstrap replications using MEGA software. The accession numbers for the sequences used here are: Human CD4 (AAH25782), mouse CD4 (AAC36010), cow CD4 (DAA29223), sheep CD4 (ABD65476), chicken CD4 (ABA55042), Xenopus CD4 (XP_002941730.2), fugu CD4-1 (NP_001072091), tetraodon CD4-1 (ABU95653), halibut CD4-1 (ACM50925), seabass CD4-1 (CAO98731), grouper CD4-1 (ADM47441), trout CD4-1 (AAY42070), salmon CD4-1 (ABZ81916), catfish CD4-1 (ABD93351), carp CD4-1 (ABD58988), catfish CD4-2 (ABD93352), halibut CD4-2 (ADP55207), tetraodon CD4-2 (ABU95652), trout CD4-2a (AY973029), salmon CD4-2a (ABZ81914.1), human LAG3 (P18627.5), mouse LAG3 (EDK99777), rat LAG3 (AAH91201), chicken LAG3 (XP_416510.2), xenopus LAG3 (XP_004915830.1), trout LAG3 (GU393008.1), medaka LAG3 (XP_004078252.1), fugu LAG3 (predicted from fugu scaffold_205).

To analyze further the evolutionary relationships of the CD4/CD4-1/CD4-2 and LAG3 molecules in jawed vertebrates, synteny analysis of the CD4 locus was undertaken and a neighbour-joining consensus tree was constructed. The synteny analysis (S5 Fig) showed some clear conservation of genes between the locus in mammals and fish (e.g. LEPREL2, GNB3, CDCA3, USP5, CLSTN3, PEX5). At least one CD4-2 gene was present in the teleost fish species examined, with two genes identified in tetraodon as well as zebrafish. In the phylogenetic tree analysis CD4, CD4-1, CD4-2 and LAG3 were found to form distinct clades (Fig 3). As expected, the zfCD4-1 molecule grouped with other known CD4-1 sequences from teleosts, whilst the two zfCD4-2’s clustered with the other teleost CD4-2 molecules, that appeared less related to CD4 of tetrapods. The respective homology of the sequences is shown in Table 1, where the similarity and identity of teleost species with known CD4-1 and CD4-2 molecules is compared to human and mouse CD4. ZfCD4-1 had highest similarity/identity to other teleost CD4-1 molecules. The two zf CD4-2 genes had 61.2% aa similarity, with next highest similarity to catfish CD4-2 (53.6% and 40%, respectively). The fish CD4-1 molecules typically have more homology to mammalian CD4 than the CD4-2 molecules (e.g. trout CD4-1 vs human CD4 is 39.5% aa similarity but trout CD4-2a vs human CD4 is 31.7%), reflecting the phylogenetic tree analysis. However, this is not as apparent in the zebrafish molecules. Analysis of the Ig domains shows that the zebrafish CD4-1 molecule has a domain structure of V-C2-V-C2 (Fig 1 (B)), identical to mammalian CD4 and other fish CD4-1 molecules. In CD4-2, since it can have a varying number of Ig domains, they differ and often do not have a clear V domain but rather a so-called intermediate (I) domain is predicted, accepting the limits of the software used. Thus, zebrafish CD4-2.1 has a predicted C2-C2-I structure, whilst zebrafish CD4-2.2 has a predicted I-C2-I-I-C2 structure.

**Table 1:** Similarity and identity analysis of CD4-related genes between mammals and teleosts.

	1	2	3	4	5	6	7	8	9	10	11	12	13
1. HumanCD4		55	19.9	18.5	17.5	20.6	18.9	23.4	17.8	21.2	16.6	23.3	18.8
2. MouseCD4	70.1		17.4	19.4	18.6	19.2	16.6	21.5	18.1	19.5	18.9	19.8	16.4
3. ZfCD4-1	37.6	36.1		20.2	18.2	34.7	15.3	29.2	14.8	43	18.7	27.3	16
4. ZfCD4-2.1	33.8	34.4	38.2		56.6	21.3	26.2	18	20	20	31.1	18.5	21.7
5. ZfCD4-2.2	32	35.5	38.3	61.2		21.7	18.8	19.9	15.4	18.8	23.6	18.1	16.3
6. TroutCD4-1	39.5	39.5	53.2	36.4	38.4		17.3	38.8	16.1	39.1	20.4	38.5	17.6
7. TroutCD4-2a	33.2	28.9	29.5	43.6	30.3	32.3		17.3	29.9	14.5	22.9	16.6	31.7
8. TetraodonCD4-1	41.6	40.6	49.2	34.3	35	57.7	30.3		17.2	35.9	20.8	48.3	17.5
9. TetraodonCD4-2	29.3	28.4	28.5	35.5	26.6	27.6	46.4	27		15.6	21.8	15.1	39.1
10. CatfishCD4-1	38.2	35.7	59.7	35.7	35.9	54.8	28.2	53.7	28.7		20.8	32.2	16.4
11. CatfishCD4-2	31.9	37.4	38.4	53.6	40	33.7	38.3	34.3	36.2	38.2		20.1	21.1
12. HalibutCD4-1	43.1	40.3	48.3	35.5	35.3	55.6	29.9	66.7	27.3	51.8	36.4		17.5
13. HalibutCD4-2	31	27.4	25.9	38	27.6	26.6	48.5	28.3	55	27.8	35.2	30.5	

**Fig 4:**
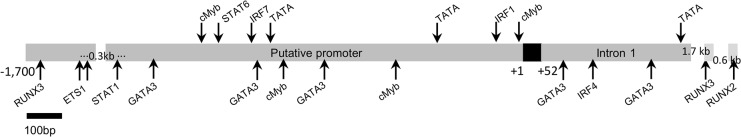
CD4-1 promoter analysis in zebrafish. In silico analysis of putative promoter and the first 1kb of the first intron of zfCD4-1. Transcription factor binding sites are shown. The sequence orientation of the transcription factor binding sites is given as upper (+ strand) and lower (- strand) annotation. cMyb = a member of the myeloblastosis family; GATA = GATA- binding factor; IRF = interferon regulatory factor family members; RUNX = Runt-related transcription factor; STAT = signal transducer and activator of transcription; TATA = cellular and viral TATA box elements. Exon 1 is coloured black.

Lastly, the zfCD4-1 5’ flanking region was examined for putative regulatory elements. Approximately 5 kbp of sequence was analyzed, including 1.7 kbp upstream of exon 1 (which is 52 bp) and the 5’ end of intron 1 (which is 13 kb). A number of transcription factor binding sites were found (Fig 4), including GATA3, cMyb and ETS binding sites, in both the putative promoter region and intron 1, and are known to be present in the human CD4-1 promoter [51]. Interestingly, Runt-related transcription factor (RUNX) binding sites, which are well known factors important for T cell (CD4/CD8 cells) development and Th cell differentiation, are present in intron 1 of zfCD4-1, as in human CD4, but absent in intron 1 of zfCD4-2.1/2.2.

Taken together the evidence suggests that in teleost fish there has been a duplication event(s) that generated two main types of CD4-related molecules, that both appear to have the potential to interact with MHCII and lck to activate Th. Whilst the function of both molecules still needs to be verified experimentally, in this study we decided to focus on the CD4-1 molecule and to develop a polyclonal anti zfCD4-1 antibody for further functional characterization of CD4-1^+^ cells in zebrafish, specifically in relation to their ability to express cytokines of adaptive immunity in an antigen-specific manner. As outlined below, FACS sorted zebrafish CD4-1^+^ cells did express the CD4-2 transcripts.

### Expression pattern of zfCD4 transcripts

**Fig 5:**
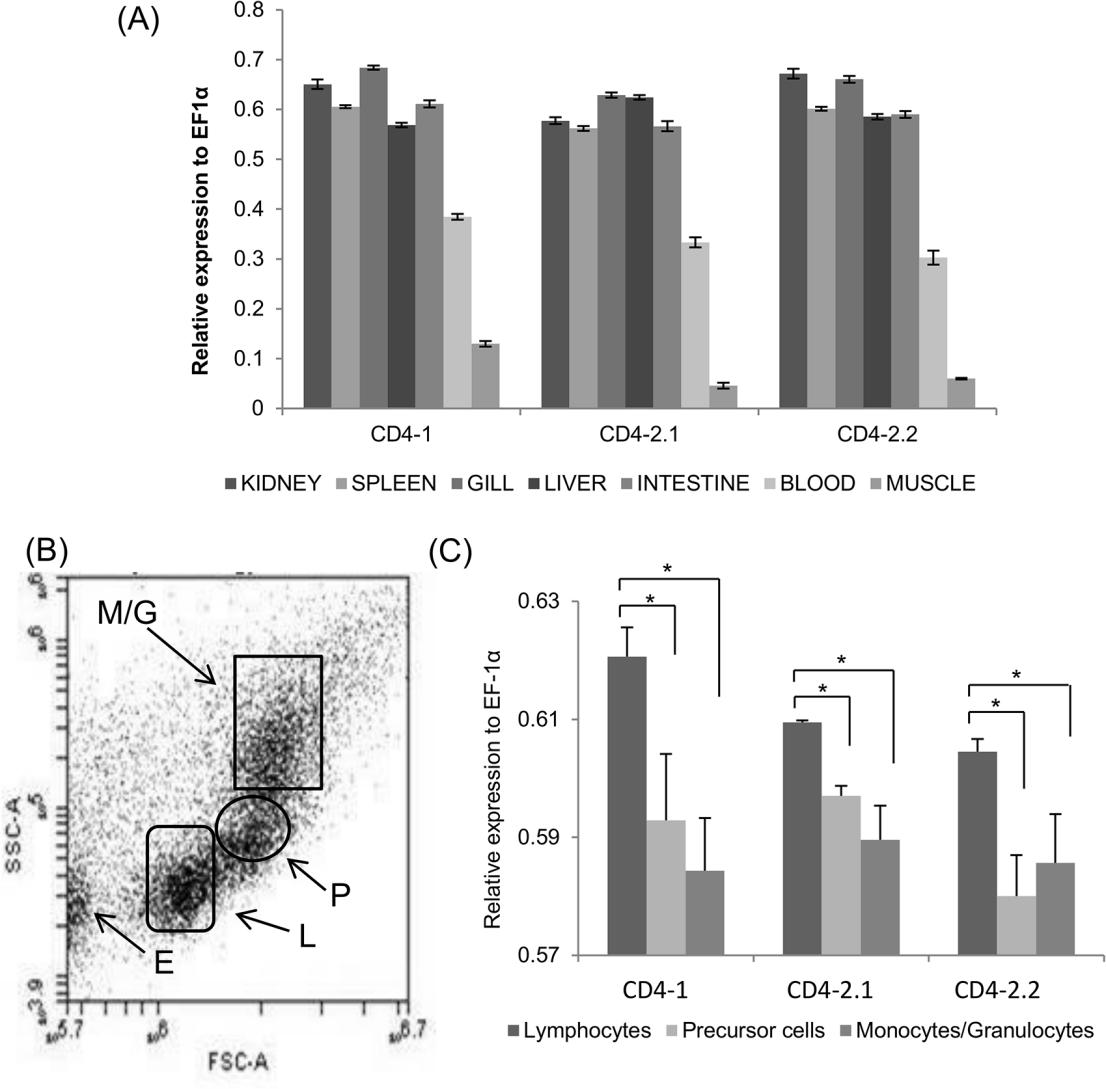
Zebrafish CD4-1/CD4-2 gene distribution. (A) Tissue/blood expression of CD4-1/CD4-2 genes in zebrafish. (B) Cell profile obtained from FACS sorted zf kidney and spleen cells. E = erythrocytes, L = lymphocytes, P = (hemopoietic) precursor cells and M/G = monocytes/granulocytes. (C) Differential expression of the zfCD4-1/CD4-2 genes within the FACS sorted populations. *, p<0.05.

QRT-PCR was undertaken to determine the transcript level of the zfCD4-1/CD4-2 genes in kidney, spleen, gill, liver, intestine, blood and muscle. All three transcripts were detected in the immune/mucosal tissues, had a relatively low expression in blood, and were hardly detectable in muscle (Fig 5 (A)). To analyze in more detail the populations that express CD4-1/CD4-2 genes in these tissues, kidney and spleen cells were pooled from 5 individuals and sorted by flow cytometry using size and complexity (Fig 5 (B)). Four clear populations were apparent within the zebrafish kidney/spleen cells; erythrocytes (lower left), monocytes/granulocytes (upper right), lymphocytes (lower right) and hemopoietic precursor cells (middle right). These terms were used based on previous studies of zebrafish leukocytes by flow cytometry [52, 53]. After sorting each population, the sorted cells were used for a second round of FACS analysis to check the purity of the sorted populations. As expected, the sorted cell populations had high purity and so were then examined for the expression of the zfCD4-1/CD4-2 transcripts by QRT-PCR (Fig 5 (C)). The results showed that the zfCD4-1/CD4-2 transcript levels were significantly higher in the lymphocyte population, with a similar, lower level of expression seen in the hemopoietic precursor cells and monocytes/granulocytes. No expression was detectable in the erythrocytes.

### Characterization of anti-zfCD4-1 serum

Two approaches were used to verify that the polyclonal antiserum generated for this investigation reacted with zfCD4-1. Firstly CHO cells were transfected with the zfCD4-1 gene, and subsequently assessed by FACS analysis and Western blotting. Secondly, Western blots were performed on zf tissue lysates.

**Fig 6:**
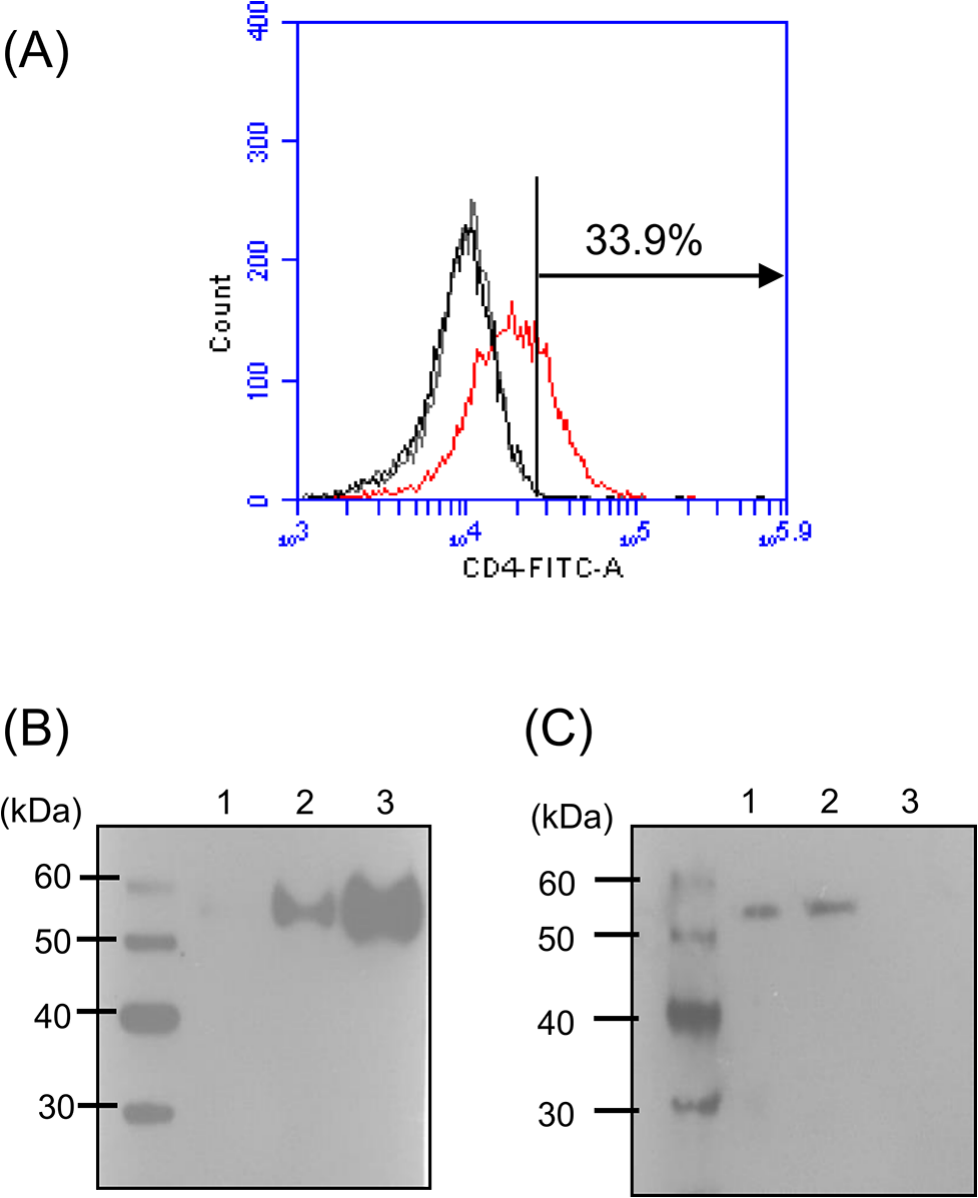
Zebrafish CD4-1 protein expression in transfected CHO cells and zebrafish tissues. (A) FACS analysis of zfCD4-1 positive cells in CHO cells transfected with zfCD4-1. Cells reacted with only secondary antibody or the pre-immune rabbit serum and secondary antibody are shown in the black and grey histogram, respectively, whilst cells incubated with zfCD4-1 polyclonal antibody and secondary antibody are shown in the red histogram. Note the increase in fluorescence intensity of the latter, as shown by a shift to the right. (B) and (C), Western blot analysis of zfCD4-1 protein. (B) Un-transfected CHO cell lysate (5 μl) (lane 1), and zfCD4-1 transfected CHO cell lysates (5μl, lane 2; 10μl, lane 3). (C), zf blood cell lysate (lane 1), kidney lysate (lane 2) and muscle lysate (lane 3). The data are representative of 3 independent experiments.

Flow cytometry of the transfected CHO cells revealed that on average 35% of cells were detected as positive for surface expression of CD4-1 with the anti zfCD4-1 polyclonal antibody, relative to transfected cells reacted with pre-immune rabbit serum or secondary Ab only, a level in line with the expected transfection efficiency of this method [54] (Fig 6 (A)). Next the CHO cells were used in Western blotting, which revealed reactivity of the anti-zfCD4-1 serum to a protein of the correct size (~52kDa) in the transfected cells but not in the untransfected cells (Fig 6 (B)), with increasing protein loaded giving a stronger reactivity. Lastly, the antibody when used in Western blots of zf blood, kidney and muscle cells, detected a single protein band of the correct size in samples rich in leukocytes (blood, kidney) but no signal was seen with muscle (Fig 6 (C)).

### Detection of zfCD4-1^+^ cells

**Fig 7:**
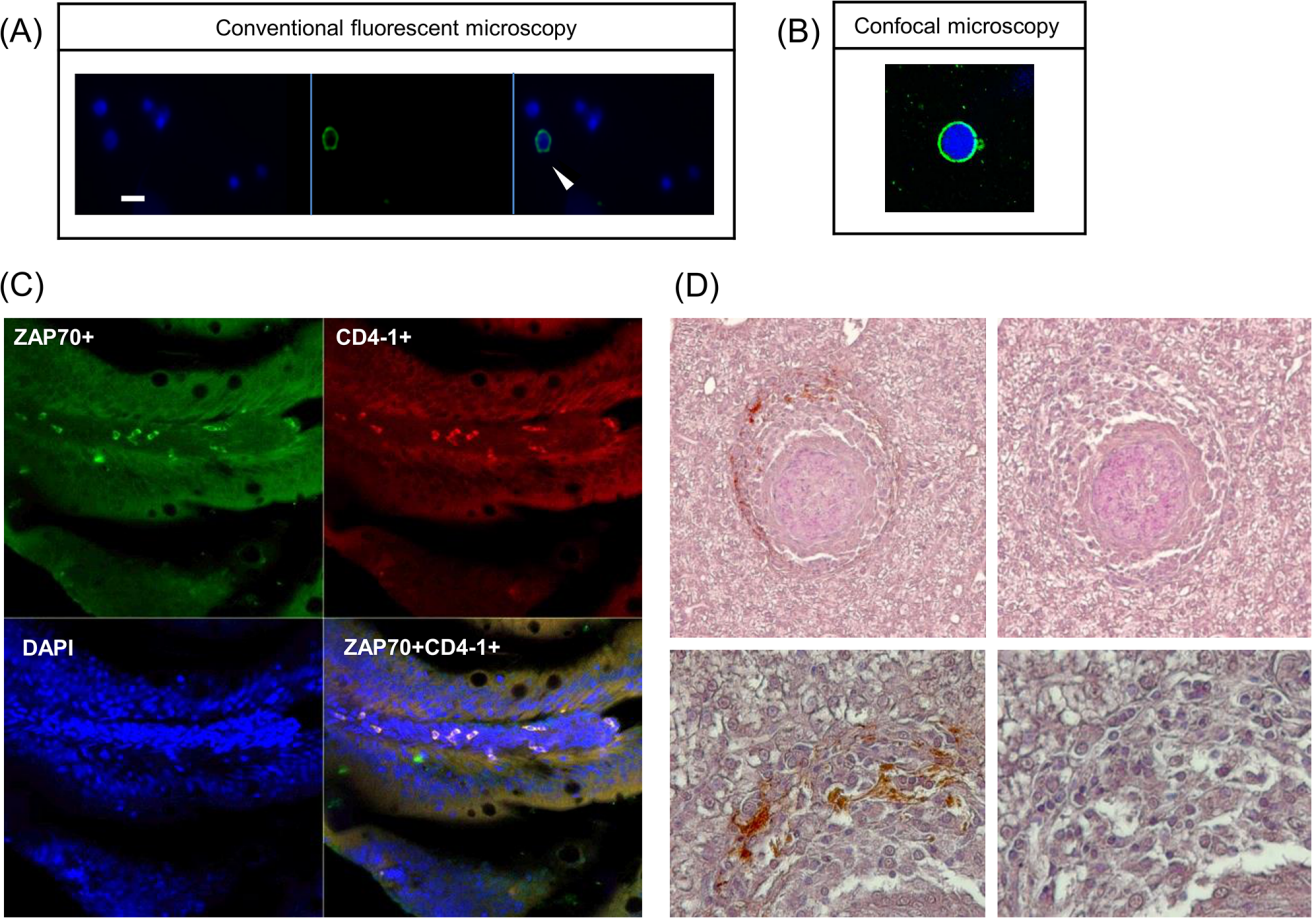
Detection of zfCD4-1^+^ cells using immunofluorescence assay and immuohistochemical assays. (A) Immunofluorescence staining of the zfCD4-1 molecule on zebrafish leukocytes. The cells stained with DAPI (blue) for counterstaining nuclei (left panel), the zfCD4-1 polyclonal followed by FITC labelled (green) secondary antibody (middle panel) and merged image (right panel) are shown. Scale bar = 10 μm). The arrow shows a cell expressing CD4 on its surface. (B) Cells from (A) were also visualized using a confocal microscope. (C) Double immunofluorescence staining of zebrafish peripheral lymphocytes in gut sections incubated with rabbit anti zfCD4-1 and mouse anti human ZAP70. ZAP70^**+**^ cells are green and CD4^**+**^ cells are red. Co-localization was confirmed by a Z-stack image analysis using Zeiss confocal microscopy. The data are representative results obtained from three independent experiments. (D) CD4-1^**+**^ cells present within the cuff of leukocytes surrounding the granulomas developed after *M*. *marinum* EspG5::Tn mutant infection of zebrafish 4 weeks earlier. Top images 200x, bottom images 400x. Left hand images used anti-CD4-1 serum, right hand images are controls.

Immune staining of zebrafish kidney leukocytes using the anti zfCD4 serum showed that the expression of zfCD4-1 was on the surface of leukocytes (Fig 7 (A)). The image from confocal microscopy (Fig 7 (B)) indicated that most CD4-1^+^ cells were lymphocyte like cells, recognized by their large nucleus surrounded by a small amount of cytoplasm. Analysis of CD4-1^+^ cells by flow cytometry revealed that in the pooled kidney/spleen suspension 12.5% of total cells were CD4-1^+^, with the highest levels seen in the lymphocyte and hemopoietic precursor gates (19–28%). In the FACS analysis using pre-immune rabbit serum as a negative control for non-specific binding, together with the secondary Ab, we established that at the dilution used (1:200) there was no impact on the fluorescence profile found (S6 Fig). Hence the threshold set up was equivalent whether using pre-immune serum or no serum as control.

A double immunofluorescence staining experiment was also performed to provide more direct evidence that some of the zfCD4-1^+^ cells in zebrafish were T cells. This included analysis of double positive cells for zfCD4-1 and ZAP70. In this experiment it was clear that double positive cells could be detected in the gut lamina propria (Fig 7 (C)), a site known to be T cell rich, suggesting that zfCD4-1^+^ T cells are found at this site.

### *M*. *marinum*-infected zebrafish develop granulomas surrounded by CD4-1^+^ cells

Previously, it was shown that an *M*. *marinum* mutant impaired in ESX-5-mediated protein secretion is hyper virulent and that zebrafish infected with the *M*. *marinum espG*_*5*_::Tn mutant develop granulomas much more rapidly and in high amounts [46]. The organization of these granulomas does not seem to differ from those induced by wild-type bacteria at a later stage. In order to analyse whether the granulomas induced in zebrafish contain, in analogy to human granulomas, CD4^+^ cells, we examined histological sections of zebrafish infected for 4 weeks with the ESX-5-deficient hyper-granulomatous *M*. *marinum espG*_*5*_::Tn mutant strain. Antibody staining demonstrated the presence of a population of CD4-1^+^ cells restricted to the outer layer and surrounding the (highly progressed) granulomas (Fig 7 (D)).

### Gene expression in sorted CD4-1^+^ and CD4-1^-^ cells

**Fig 8:**
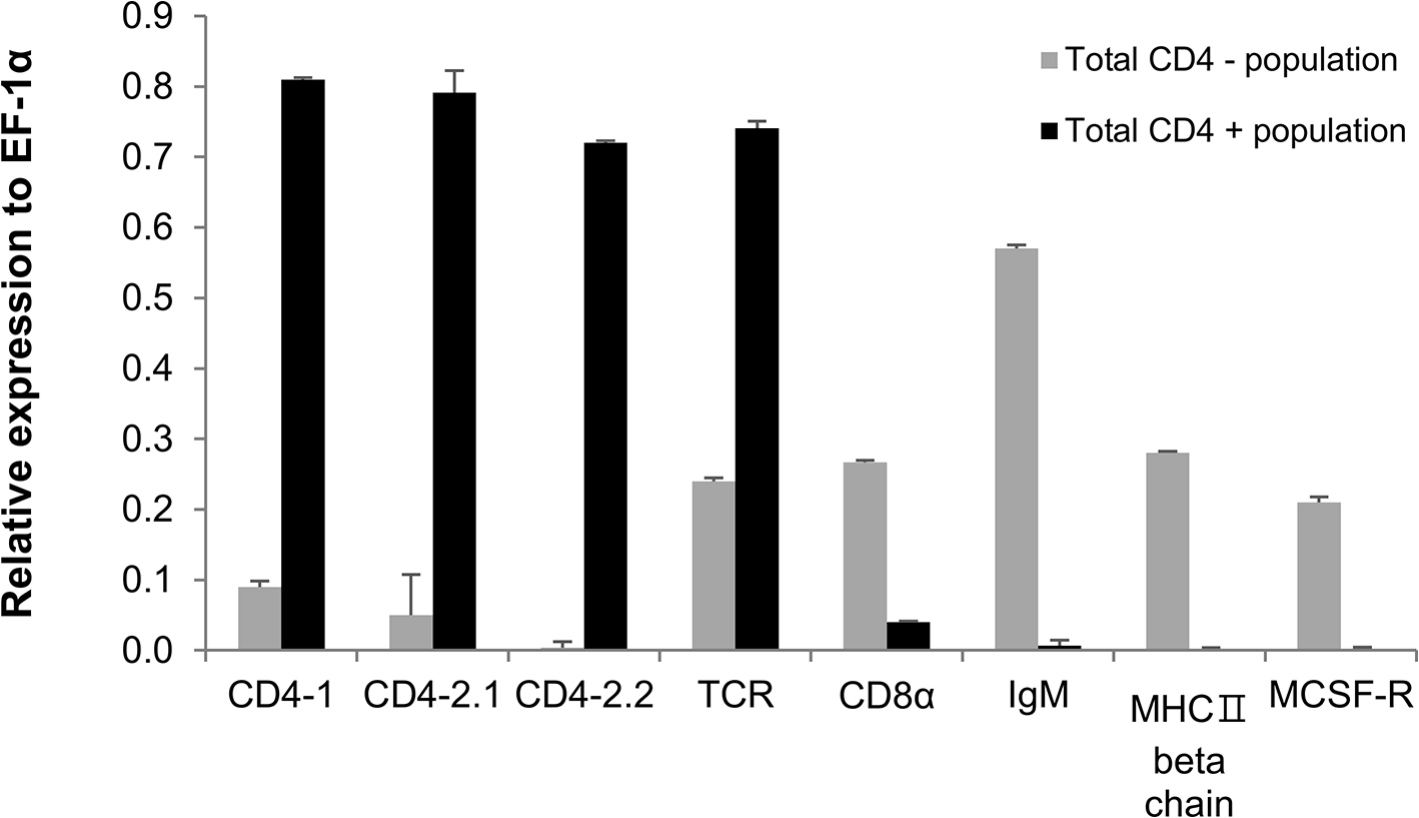
QRT-PCR analysis of various cell markers for different leukocyte populations expressed by CD4-1^+^ and CD4-1^-^ cells. Each bar represents the mean of three independent measurements expressed relative to EF-1α.

To compare the gene expression profile of sorted CD4-1^+^ and CD4-1^-^ cells we analysed a number of key cell surface markers by real time PCR (Fig 8). These results showed that there was a marked difference between the two populations in terms of their expression of CD4-1, CD4-2.1, CD4-2.2, which were almost exclusively found in the CD4-1^+^ population, and CD8, IgM, MHC IIβ and MSCF-R which were almost exclusively found in the CD4-1^-^ population. The TCRα chain expression was present in both populations, although higher in the CD4-1^+^ cells, possibly as a consequence of the CD4-1^-^ cells being a more heterogeneous population.

### Antigen-induced expression of cytokines of adaptive immunity in CD4-1^+^ cells sorted from the lymphoid population

**Fig 9:**
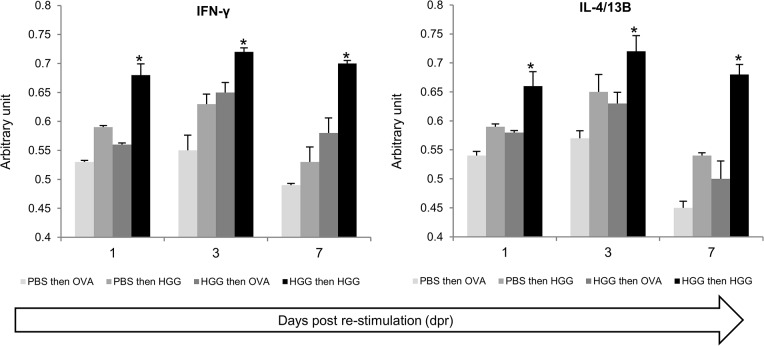
The transcript levels of IFN-γ and IL-4/13B in kidney of zebrafish given antigen for the first time, or following boosting with specific or non-specific antigen, were determined by QRT-PCR. Each bar represents the expression relative to EF-1α. PBS then OVA: fish given PBS and subsequently primed with OVA. PBS then HGG: fish given PBS and subsequently primed with HGG. HGG then OVA: fish primed with HGG and subsequently boosted with OVA. HGG then HGG: fish primed with HGG and subsequently boosted with HGG. The mean values are presented for 6 individual fish. *, significantly different (P<0.05) against PBS injected fish.

In a first experiment, the relative expression level of IFN-γ, IL-4/13A and IL-4/13B to a housekeeping gene was determined in four groups of zebrafish that were immunised with HGG or OVA, with or without prior immunisation with HGG (Fig 9). Zf that were primed and boosted with HGG showed the highest level of induced expression of IFN-γ and IL-4/13B relative to the groups that received a primary immunisation with HGG or OVA or that were boosted with OVA (after HGG priming) at all times points examined (although more marked at days 3 and 7 post boosting). Interestingly, no significant induction of IL-4/13A was detected at any of the timings/samples analysed (i.e. no positive fluorescence level was detected). In comparison to gene expression levels in fish given PBS alone at the time of the second injection, only the HGG boosted fish showed significantly elevated cytokine levels (at all timings).

**Fig 10:**
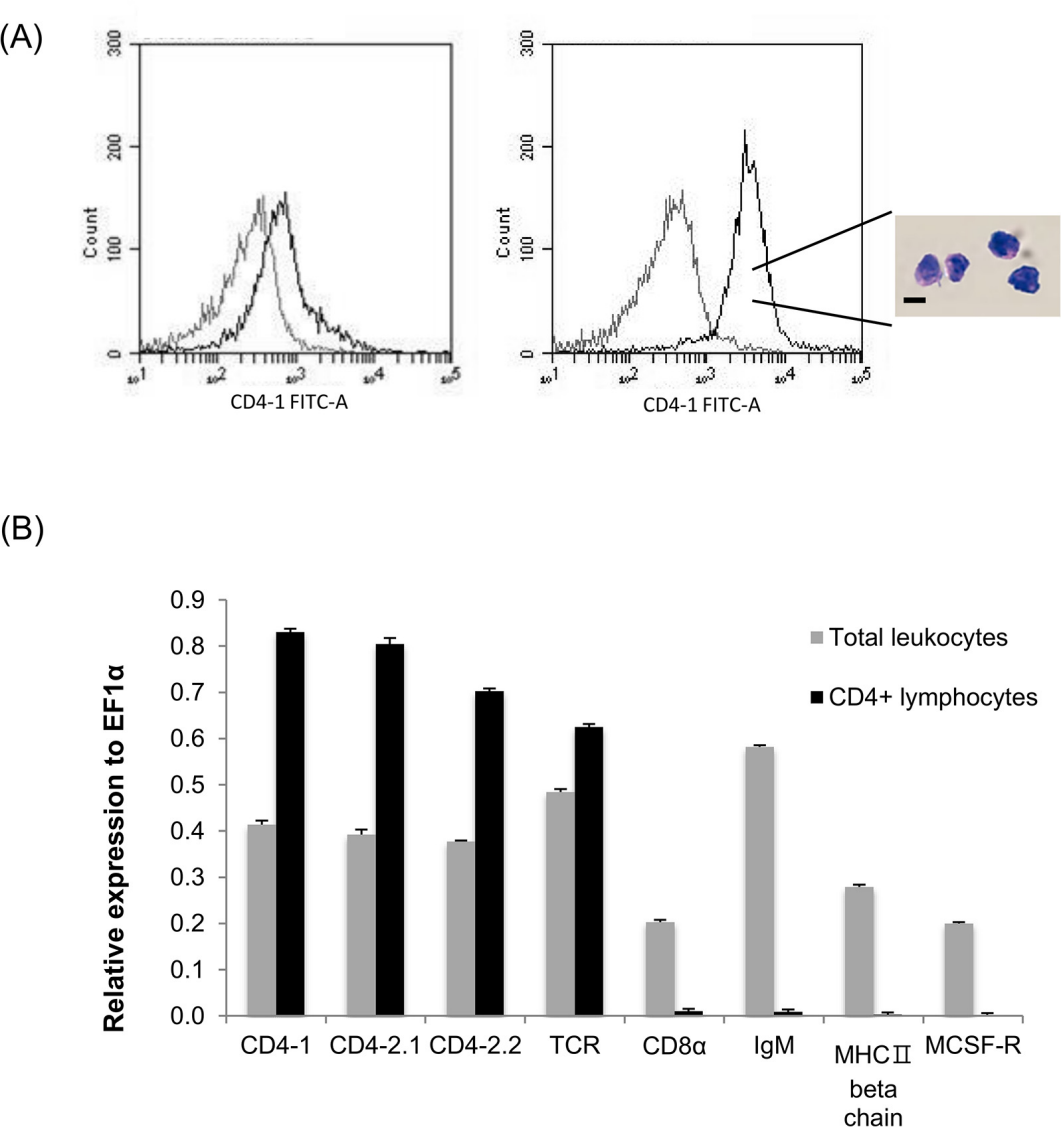
ZfCD4-1 expression from the sorted CD4-1^+^ cells. (A) FACS plot showing CD4-1^**+**^ cells from the lymphocyte gate (see Fig 5 (B)). In the overlay histogram the CD4-1^**+**^ lymphocyte population is indicated with a black line whilst control cells stained with secondary Ab only are shown with a grey line. The left histogram shows unsorted cells whilst the right one shows FACS purified CD4-1^**+**^ cells. H&E staining shows the morphology of the sorted CD4-1^**+**^ lymphocytes. Scale bar = 5μm. (B) Transcript expression of different cell marker genes, as assessed by QRT-PCR in FACS purified CD4-1^**+**^ cells sorted from the lymphocyte gate and unsorted cells. Each bar represents the mean of three independent measurements expressed relative to EF-1α.

**Fig 11:**
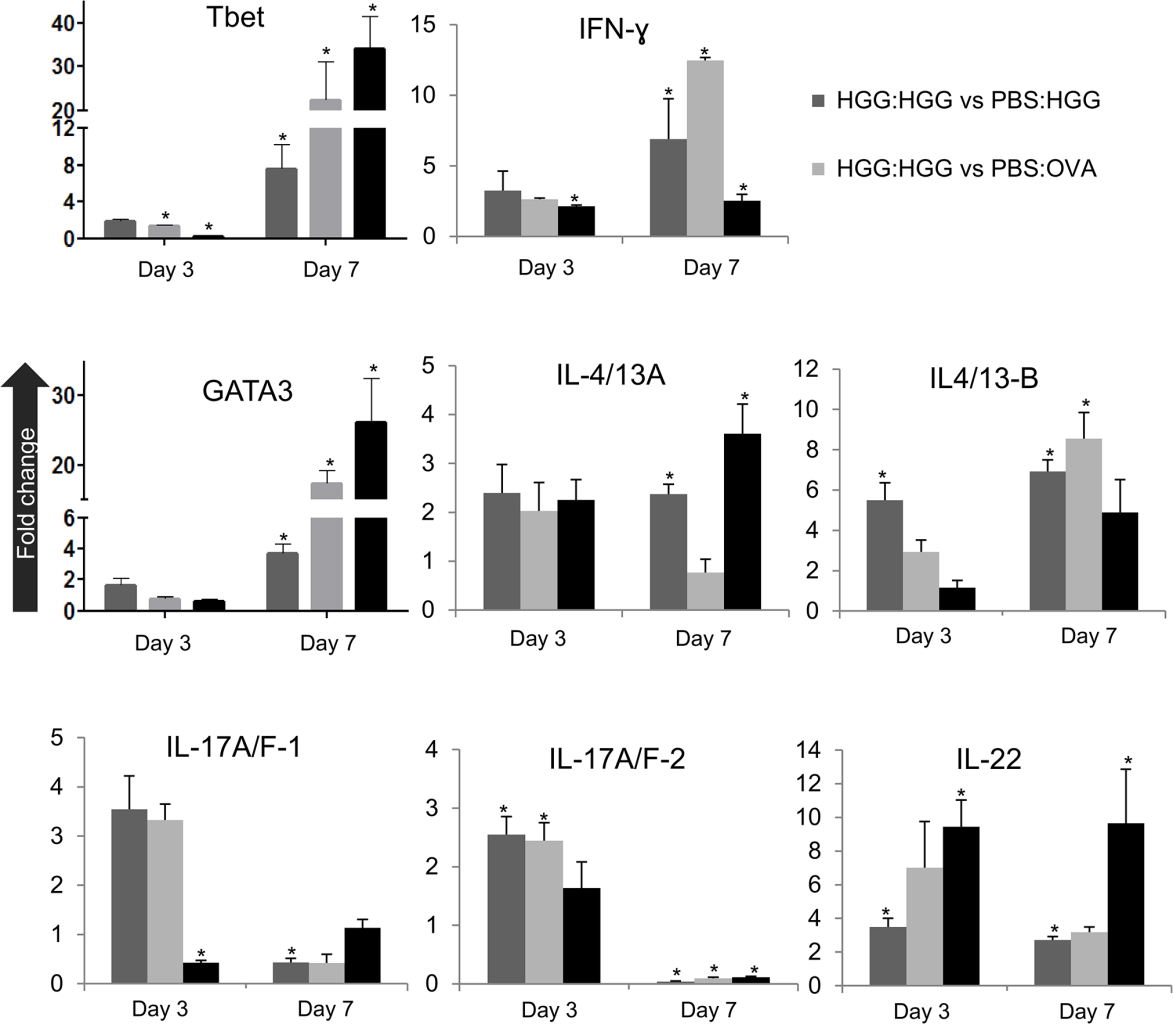
The transcript levels of different cytokines in CD4-1^+^ sorted lymphocytes from kidney and spleen of zebrafish given antigen for the first time, or following boosting with specific or non-specific antigen, were determined by QRT-PCR. Data were first normalized to the expression level of EF-1α, and then expressed as a fold change by comparing the transcript levels in samples from fish primed and boosted with HGG (HGG:HGG), in comparison to fish initially given PBS followed by antigen for the first time (e.g. PBS:HGG, or PBS:OVA), or fish given HGG initially followed by OVA (HGG:OVA). The values presented are means of 4 replicates (of 5 pooled fish). *, P<0.05 between the two groups compared in each graph.

These promising results prompted a second experiment to determine if the antigen-induced increases in adaptive cytokine expression could be detected in CD4-1^+^ lymphocytes. To this end the experiment was repeated, with a focus on days 3 and 7 post-boosting, and with pooling of tissue (i.e. kidney and spleen) from 5 fish (with four biological replicates) to obtain enough cells from FACS sorting of CD4-1^+^ cells from the lymphocyte gate for gene expression analysis (Fig 10 (A)). The sorted CD4-1^+^ cells from the lymphocyte gate showed a typical lymphocyte morphology when stained with H&E, and were ~6–7 μm in diameter with a large nucleus and small cytoplasmic rim (Fig 10(A)). As can be seen in Fig 10 (B), transcript analysis for a variety of cell surface markers showed that these cells expressed CD4-1, CD4-2.1, CD4-2.2 and TCRα but were negative for IgM (μ chain), MHC IIβ chain, CD8α and MCSF-R, that were all expressed in the unsorted cells. In this experiment the cytokine and transcription factor data are presented as a fold change of the results from the HGG boosted fish relative to the other three groups (i.e. values above 1 indicate the response is higher in the antigen boosted fish). As in the first experiment, expression of IFN-γ and IL-4/13B in CD4-1^+^ cells sorted from the lymphocyte gate was again found to be significantly higher in the cells from HGG boosted fish compared to cells from fish given HGG or OVA once only, or boosted with OVA following HGG priming (Fig 11), especially at day 7. Further genes were also examined in this experiment, with a focus on genes relevant to effector Th cell responses. We found that induction of Tbet and GATA3, the master transcription factors for Th1 and Th2 cells respectively, was correlated with the peak induction of IFN-γ and IL-4/13B, with significant increases seen at day 7 post-boosting, with highest levels found relative to the HGG primed fish that were subsequently administered OVA. A small induction of IL-4/13A was also detected in this experiment in two of the three comparisons. IL-22 expression was also increased significantly in the HGG boosted fish vs the HGG only fish or fish boosted with OVA, at both timings. However, IL-17A/F1 and IL-17A/F2 were increased to some extent at day 3 (2–3 fold) but not at day 7, and in fact IL-17A/F2 expression was down-regulated at day 7 in the HGG boosted fish compared to cells from fish only primed with either antigen or boosted with OVA.

## Discussion

Th responses are well studied in mammals but very little is known about when such responses arose during the evolution of adaptive immunity, or indeed whether Th cells equivalent to Th subtypes, such as Th1, Th2 and Th17 exist in lower vertebrates. This is despite the characterization of T cells in jawed fish, and the presence of CD4-related molecules, MHC II and many relevant cytokine genes in the euteleostomi. CD4 molecules contribute to T cell activation by influencing the activity of the TcR-antigen-MHC II interaction and recruiting the co-receptor-bound cytoplasmic protein tyrosine kinase (lck) to the TcR signaling complex [55, 56]. Following activation of T cells they proliferate and differentiate into the known mammalian subpopulations and release distinct cytokine repertoires that can activate antimicrobial mechanisms appropriate to the pathogen encountered. In the context of the evolution of Th responses it is known that in bony fish CD4-1^+^ cells can express cytokines relevant to adaptive immunity after stimulation with mitogens and PAMPs [28] and that CD4-1^+^ cells can undergo proliferation when stimulated with specific antigen in vitro [27, 57]. However, no specific induction of cytokine expression has been reported in CD4-1^+^ (or CD4-2^+^) cells following antigen exposure, and hence it is unknown whether Th equivalents exist in fish despite evidence for their role in protective responses [29, 30]. This is critical to the discussion of when different Th subpopulations evolved and whether it is possible to manipulate Th subpopulations in fish during vaccination, as a desirable means to improve fish health in aquaculture. Thus, in this study we explored whether purified CD4-1^+^ lymphocytes from fish can be shown to undergo antigen-induced expression of cytokines considered typical of the major Th subsets characterized in mammals.

We chose zebrafish as the model, since many of the cytokine genes we wanted to monitor are already well characterized (IFN-γ, IL-4/13, IL-17, etc.) and there are many established antigens that are known to stimulate specific immunity in cyprinids [33, 58]. When we examined the zebrafish CD4 locus, we found that three CD4-related genes were present adjacent to each other on chr16, which after bioinformatics analysis we termed zfCD4-1 zfCD4-2.1 (or zfCD4rel1) and zfCD4-2.2 (or zfCD4rel2). This is in line with other teleost species, where both CD4-1 and CD4-2 molecules are present [20, 24, 25], although the number of the latter is variable and likely due to extra gene duplication in some species. zfCD4-1 had 4 predicted Ig domains, typical of CD4 in other vertebrate species, two of which are variable Ig domains (D1 and D3) and two are constant Ig domains (D2 and D4) [43]. An lck motif was also present in the C-terminal intracellular region, and transcription factor binding sites for GATA3, cMyb and ETS were found in the putative CD4-1 promoter and intron 1, known to be present in the human CD4 promoter [51], and to be important for modulating CD4 expression. Interestingly RUNX sites were also found. RUNX proteins have a critical function in T cell development [59] and contribute to the differentiation of naïve CD4^+^ T cells into T cell subsets. In contrast, the zfCD4-2 molecules had 3 (zfCD4-2.1) or 5 (zfCD4-2.2) Ig domains, and an lck site was present in the intracellular domain of zfCD4-2.1 (there is no intracellular domain in zfCD4rel2). Again, this is in line with findings in other teleost fish species [18], but with the additional observation that RUNX regulatory elements appeared missing from the zfCD4-2.1 and -2.2 5’flanking region/intron 1. Our modelling of the potential to bind to MHCII indicated that both CD4-1 and CD4-2 could do this, as seen previously in seabass for CD4-1 [21], although the aa residues predicted to be involved in this interaction differ between the zf molecules.

At the transcript level the zfCD4-1 and zfCD4-2 genes were highly expressed in various lymphoid tissues, including liver which contains immune cells (T cells, B cells, myeloid cells) and has an immune function in fish [60, 61], with no clear differences in expression level between the genes when analyzing total tissue RNA. This result is similar to earlier findings from other fish species [20, 21, 62, 63]. In addition, previous work in our lab using whole mount *in situ* hybridization (WISH) with a CD4-1 antisense RNA probe demonstrated that the zfCD4-1 was also expressed within the developing thymus in zebrafish embryos 4 days post fertilization [64]. The expression of CD4-1/CD4-2 in cells sorted by flow cytometry was also studied, and showed that these genes are more highly expressed in cells from the lymphocyte gate, again in agreement with previous findings in fish [18]. That some level of transcript expression was detectable in the precursor and monocyte/granulocyte populations was expected and will be discussed below in the context of the protein expression.

To analyse CD4-1 cell surface expression, a rabbit polyclonal anti zfCD4-1 serum was produced and shown to bind zfCD4-1 in transfected CHO cells. It should be noted that the relatively high fluorescence level for the non-transfected cells is likely due to the intrinsic cellular fluorescence (auto-fluorescence) of cultured mammalian cells [65]. When used in Western blot analysis of cell lysates from blood and kidney, it detected a protein of the correct size in these lymphoid tissues but not with muscle. This also shows that the zfCD4-1 antibody detects only the zfCD4-1 protein and not the CD4-2 proteins. When used in immunofluorescence it detected surface staining on kidney leukocytes, in lymphocyte-like cells, as seen with kidney leukocytes of ginbuna [27]. In addition, in co-localization studies with antisera to another T cell marker, ZAP70, double positive cells could be detected in the gut, a tissue rich in T cells [66, 67], and is the first demonstration of the co-existence of these molecules in any fish species. The zfCD4-1 antibody was also used in FACS analysis to determine the numbers of CD4-1^+^ cells present in kidney/spleen cell suspensions. This showed that CD4-1^+^ cells represented 12.5% of the total cell population, whilst in gated populations they ranged from 19–28% in the lymphocyte and hemopoietic precursor fractions. Toda *et al* have previously reported the numbers of CD4-1^+^ cells in ginbuna [27], where 11% of splenocytes, 23% of head-kidney cells and 19.5% of trunk-kidney cells were positive. Interestingly, our study showed there were some cells positive for zfCD4-1 in the monocyte/granulocyte gate, in accord with the detection of CD4-1 transcript in these cells (albeit at a lower level than in the lymphocyte fraction). This suggests that it is not only T cells that express CD4-1 in fish, and is in agreement with human studies where in both healthy uninfected volunteers and in HIV-infected patients, CD4 can also be found on monocytes, macrophages and neutrophils [68, 69]. To analyse whether CD4-1^+^ cells are involved in the responses to infection, a *M*. *marinum* model was investigated, whereby the developing granulomas were examined for the presence of CD4-1^+^ cells. Such cells were clearly seen in the leukocyte cuff that surrounds the granulomas that are a hallmark of this disease. Future studies will focus on the cytokines associated with this site. Lastly, we examined the gene expression profile of surface markers associated with different leukocyte types in sorted CD4-1^+^ and CD4-1^-^ cells. As expected we found that there was a clearly enhanced expression level of CD4-1, but also CD4-2.1 and CD4-2.2 in the CD4-1^+^ cells, suggesting these molecules are likely co-expressed. Expression of the B cell and macrophage markers (MHC IIβ, MCSF-R) was absent from the CD4-1^+^ cells but present in the CD4^-^ cells. Lastly, TCRα chain expression was present in both populations.

The next goal was to confirm that enhanced cytokine expression could be detected in the antigen-restimulation model chosen for this study. Following *in vivo* priming and boosting with the antigen, kidney cells were isolated and cytokine expression was analysed. Indeed it was possible to detect good increases in transcript levels of a key Th1 and Th2 type cytokine, IFN-γ and IL-4/13B, respectively, with maximal increases typically seen at day 3 or day 7 post-boosting. These responses were significantly higher than in cells from fish injected with PBS or antigen for the first time (HGG or OVA), or primed with HGG and then given OVA. Indeed, the responses seen in the cells from fish given a primary immunisation with HGG or OVA, or boosted with OVA (in HGG primed fish), were not significantly different to those in PBS injected fish, and confirms that boosting is needed to get clear cytokine profiles. Curiously there was no increase in IL-4/13A in this experiment, the second Th2-type cytokine present in fish, which is in line with the results of Yamaguchi *et al* demonstrating significant enrichment of IL-4/13B but not IL-4/13A in KoThL5 cells stimulated with PHA, which show some features in common with mammalian Th2 cells including expression of TcR genes, CD4-1 and GATA3 [47, 70]. Having established the restimulation model gave detectable cytokine transcript levels, a second experiment was performed to examine whether the specific cytokine induction could be linked to CD4-1^+^ lymphocytes since some level of CD4-1 expression (at least of the transcript) was apparent in other cell types. Emphasis was placed on sorting the cells to high purity to avoid contamination with CD4-1^-^ cells, and cells were only collected from the lymphocyte gate to avoid collecting other (non-lymphoid) cells that may express CD4-1 on their surface (although it is still to be proven such cells exist). The purified CD4-1^+^ cells had a lymphocyte-like morphology and were found to express CD4-1, CD4-2.1, CD4-2.2 and TCRα transcripts but not CD8, and were negative for the B cell markers Igμ and MHC IIβ, and the macrophage marker MCSF-R, showing the sorting had fractionated the cells as expected. Again clear increases in key cytokine transcript levels were detectable in the sorted cells, with Tbet and IFN-γ in particular showing large increases at day 7 post-HGG boosting relative to responses in the groups given a single antigen injection (HGG or OVA) or that were boosted with OVA (after HGG priming). IL-4/13A and IL-4/13B also showed significant increases relative to the other groups, again with highest increases seen at day 7, associated with a significant increase in GATA3 expression. IL-22 was also increased upon HGG boosting compared to fish given HGG for the first time or that were boosted with OVA. In contrast only small increases in IL-17A/F expression were seen, with maximal increases at day 3 and in the case of IL-17A/F2, significant inhibition at day 7 were seen relative to the other groups. Past studies with HGG in carp and zebrafish have shown it is an excellent immunogen for specific antibody production [33, 71] and it is considered a T cell-dependent antigen in fish [72]. The present data suggest that if the types of Th responses in fish are similar to mammals, then Th1 and Th17 cells are not induced concurrently, and potentially one may inhibit the other.

In conclusion, this study demonstrates the antigen-stimulated induction of cytokine responses in CD4-1^+^ lymphocytes in fish for the first time. Such cells are potentially the equivalents of Th cells in homeotherms, although it is still to be established if subpopulations exist that produce restricted cytokine repertoires induced by different stimuli. These cells also express the zfCD4-2 transcripts, and if this results in protein expression then it is possible that CD4-1/CD4-2 double positive cells will be present. Whilst the possible functional interaction between these molecules awaits further study, the present results suggest that Th cells likely existed in early vertebrates prior to the divergence of bony fish from the main vertebrate lineage.

## Supporting Information

S1 Fig Sequence alignment of zebrafish, mouse and human MHC II_A. (PDF)

S2 Fig Sequence alignment of zebrafish, mouse and human MHC II_B. (PDF)

S3 Fig Validation of the ZAP70 polyclonal antibody using Western blot analysis of zebrafish leukocyte lysates. (PDF)

S4 Fig Multiple sequence alignment of zfCD4 proteins with other CD4 and LAG3 homologues. (PDF)

S5 Fig Synteny analysis of the CD4 locus in selected teleost fish species and in mammals (human). (PDF)

S6 Fig Flow cytometry analysis of zebrafish leukocytes reacted with rabbit pre-immune serum and secondary Ab, compared to using secondary Ab only. (PDF)

S1 Table Primers used in cDNA synthesis and RACE PCR for zfCD4 genes. (PDF)

S2 Table Primers used in real time PCR for gene expression in zebrafish. (PDF)
