# Correction: First Demonstration of Antigen Induced Cytokine Expression by CD4-1^+^ Lymphocytes in a Poikilotherm: Studies in Zebrafish (*Danio rerio*)

**DOI:** 10.1371/journal.pone.0169149

**Published:** 2016-12-21

**Authors:** Sohye Yoon, Suman Mitra, Cathy Wyse, Ayham Alnabulsi, Jun Zou, Eveline M. Weerdenburg, Astrid M. van der Sar, Difei Wang, Christopher J. Secombes, Steve Bird

The CD4-1-R primer sequence is listed incorrectly in S2 Table. The correct primer sequence is: 5'CTGGTCGGTCTTAAATGAAACT3'. Please see the correct S2 Table here.

## Supporting Information

S2 Table Primers used in real time PCR for gene expression in zebrafish. (DOCX)
