# Supplementary material for: First Demonstration of Antigen Induced Cytokine Expression by CD4-1+ Lymphocytes in a Poikilotherm: Studies in Zebrafish (Danio rerio)
Source: PLoS One. 2015 Jun 17;10(6):e0126378. doi: 10.1371/journal.pone.0126378 (PMC4470515; doi:10.1371/journal.pone.0126378)
Supplement: S2 Fig — (PDF) [file pone.0126378.s002.pdf]

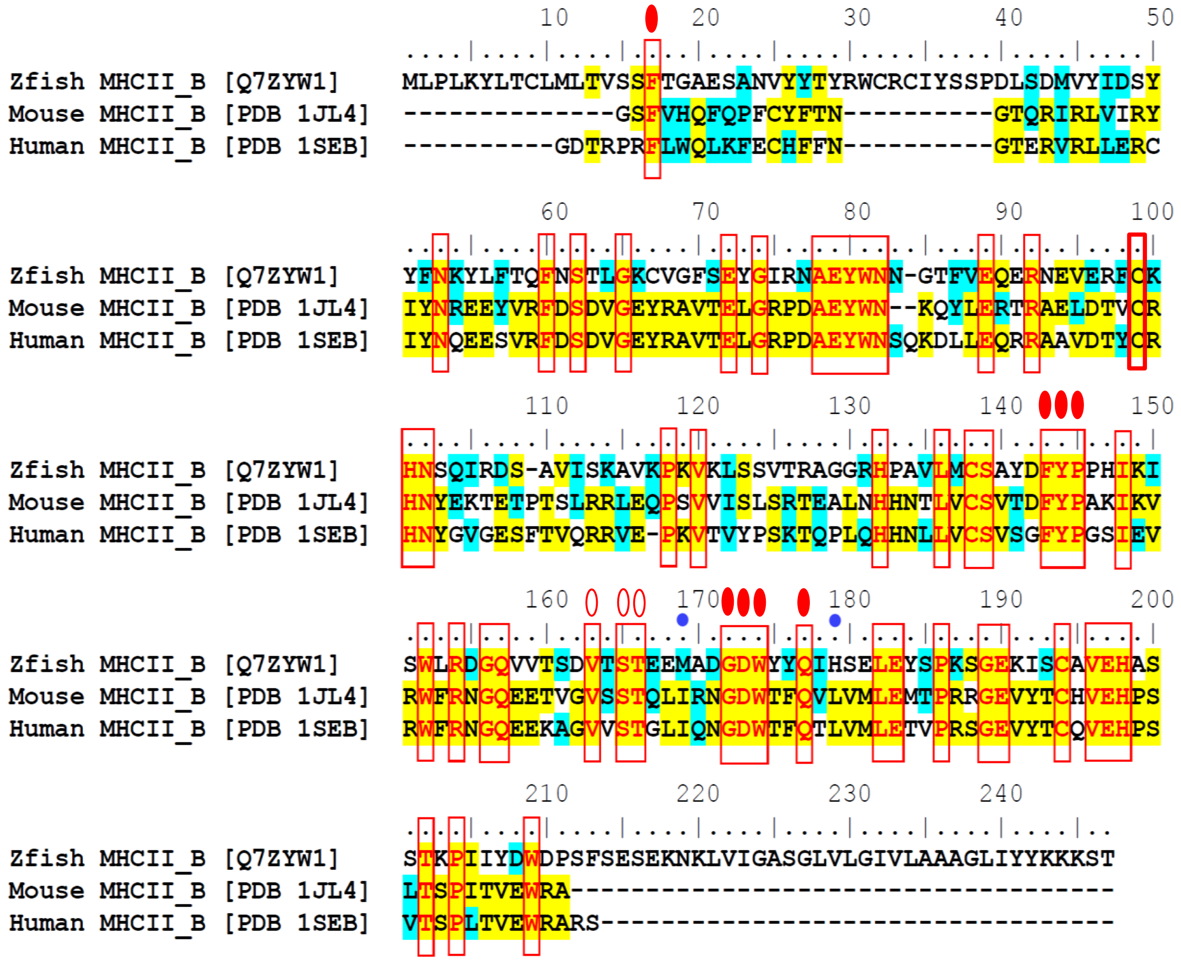

Figure S2. Sequence alignment of zebrafish, mouse and human MHC II_B. Yellow: identical; Cyan: similar; Boxed: identical for all three; Blue dots: observed residues which make contact with the CD4 molecule in X-ray structure (PDB: 1JL4). Red ovals: conserved residues at the interface between MHCII_A and MHCII_B. Empty ovals: conserved residues that interact with CD4 molecules.
