# Supplementary material for: First Demonstration of Antigen Induced Cytokine Expression by CD4-1+ Lymphocytes in a Poikilotherm: Studies in Zebrafish (Danio rerio)
Source: PLoS One. 2015 Jun 17;10(6):e0126378. doi: 10.1371/journal.pone.0126378 (PMC4470515; doi:10.1371/journal.pone.0126378)
Supplement: S3 Fig — (PDF) [file pone.0126378.s003.pdf]

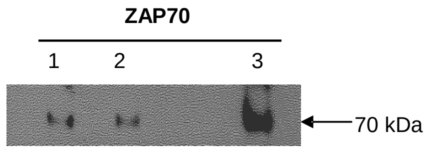

Figure S3. Validation of the ZAP70 polyclonal antibody using Western blot analysis of zebrafish leukocyte lysates. Specificity of the anti-ZAP70 antibody was confirmed by detection of a 70 kDa protein. Lanes 1, 2 and 3 represent different amounts of total protein (5ug, 10ug and 15ug), respectively.
