# Supplementary material for: First Demonstration of Antigen Induced Cytokine Expression by CD4-1+ Lymphocytes in a Poikilotherm: Studies in Zebrafish (Danio rerio)
Source: PLoS One. 2015 Jun 17;10(6):e0126378. doi: 10.1371/journal.pone.0126378 (PMC4470515; doi:10.1371/journal.pone.0126378)
Supplement: S4 Fig — (PDF) [file pone.0126378.s004.pdf]

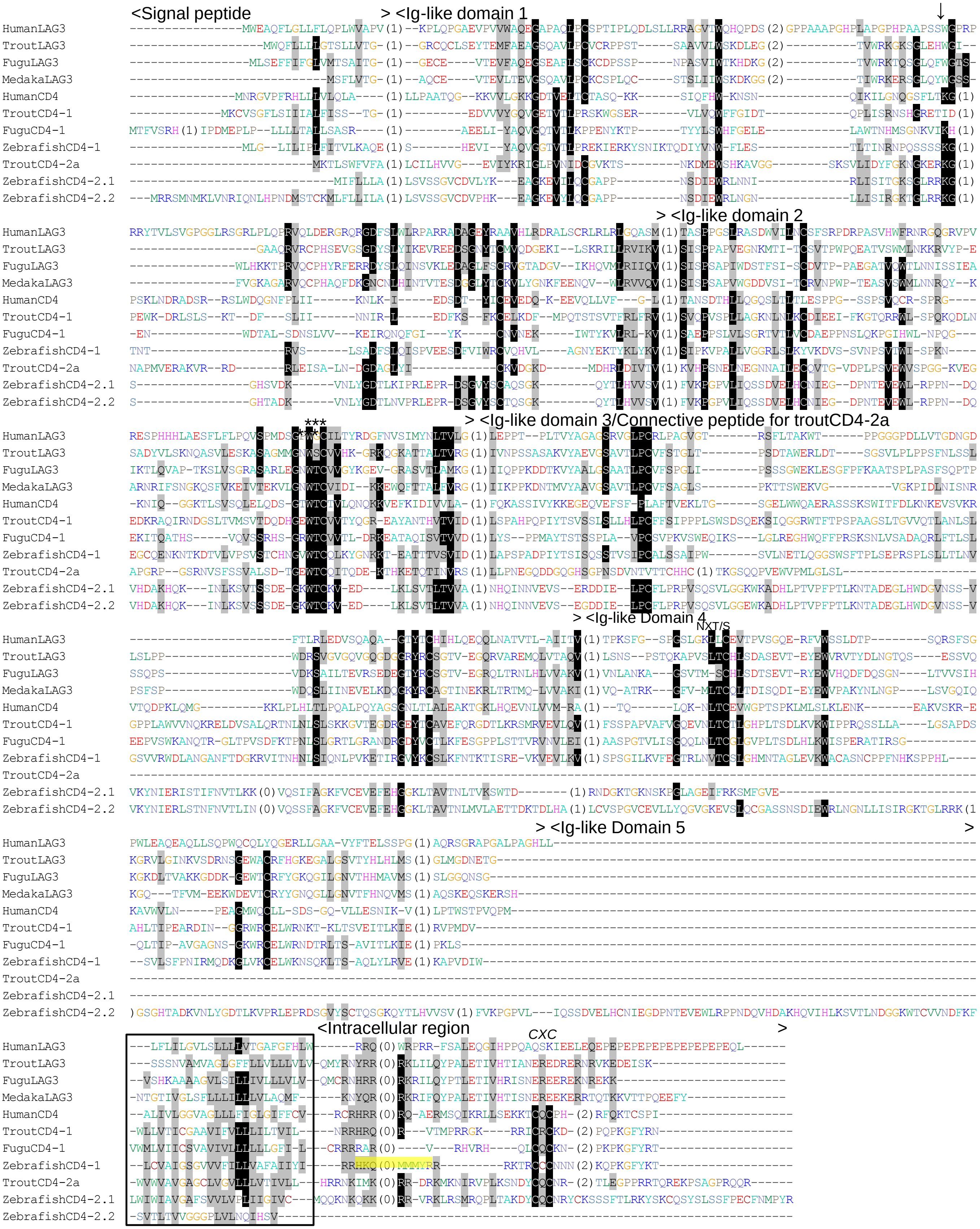

Figure S4. Multiple sequence alignment of zfCD4 proteins with other CD4 and LAG3 homologues. The lck binding site (CXC) is labelled above the sequence in italics and Lys46 for CD4 is indicated with an arrow above the sequence. Other conserved regions include the WTC, marked with asterisks, and N-linked glycosylation sites (NXT/S), labelled above the sequence. The transmembrane domain is boxed and predicted amphipathic alpha-helix motif is highlighted in yellow. (x) indicate intron position with intron phase.
