# Supplementary material for: First Demonstration of Antigen Induced Cytokine Expression by CD4-1+ Lymphocytes in a Poikilotherm: Studies in Zebrafish (Danio rerio)
Source: PLoS One. 2015 Jun 17;10(6):e0126378. doi: 10.1371/journal.pone.0126378 (PMC4470515; doi:10.1371/journal.pone.0126378)
Supplement: S5 Fig — (PDF) [file pone.0126378.s005.pdf]

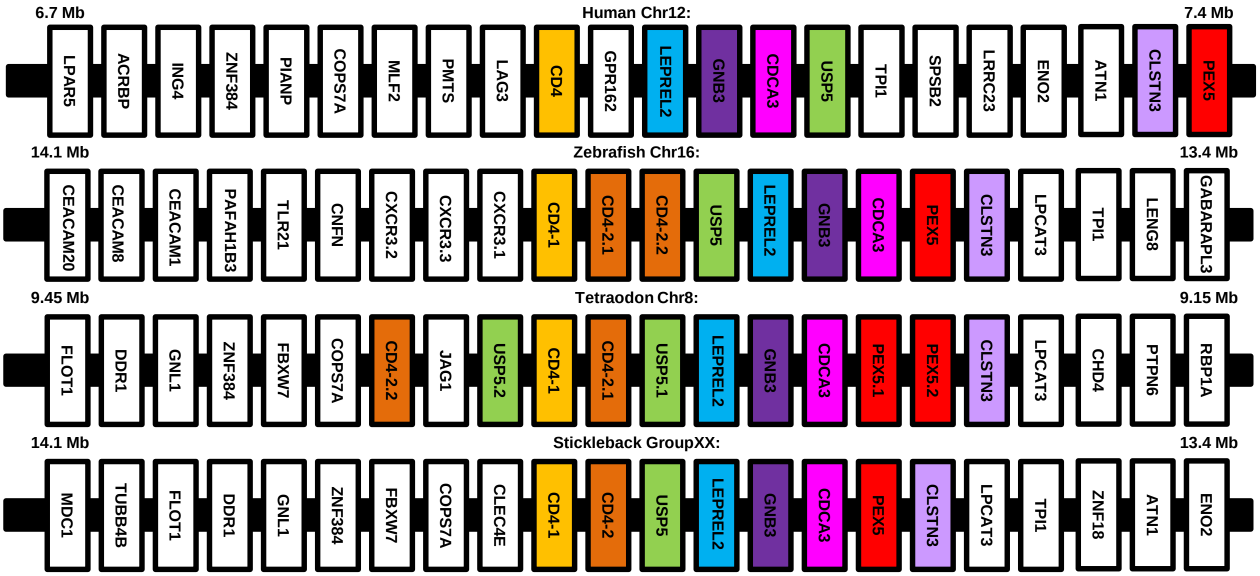

Figure S5. Synteny analysis of the CD4 locus in selected teleost fish species and in mammals (human). Note the common genes (coloured) between the different species).
