# Supplementary material for: First Demonstration of Antigen Induced Cytokine Expression by CD4-1+ Lymphocytes in a Poikilotherm: Studies in Zebrafish (Danio rerio)
Source: PLoS One. 2015 Jun 17;10(6):e0126378. doi: 10.1371/journal.pone.0126378 (PMC4470515; doi:10.1371/journal.pone.0126378)
Supplement: S6 Fig — (PDF) [file pone.0126378.s006.pdf]

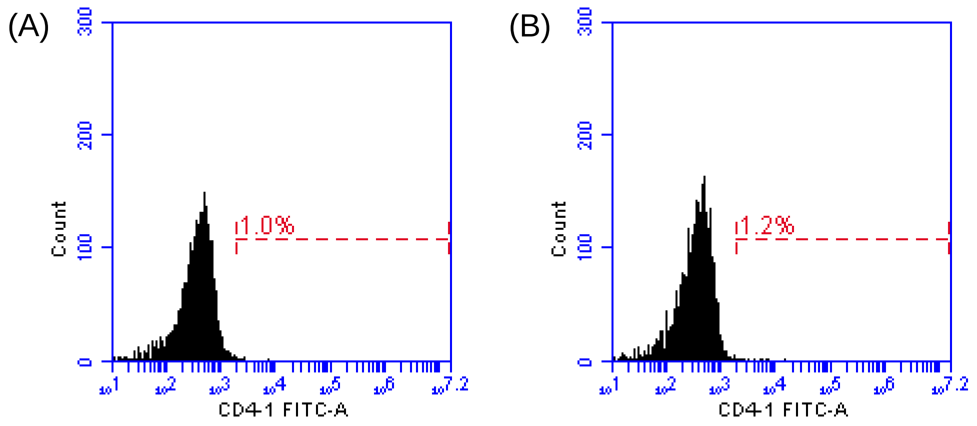

Figure S6. Flow cytometry analysis of zebrafish leukocytes using isotype control compared to cells stained only secondary Ab. (A) FITC conjugated anti rabbit secondary antibody was used here and bar was set to allow for 1 % auto-fluorescence cells (false-positives). (B) 1in 200 dilution of pre-immunized rabbit sera was used as an isotype control and the bar setting strategy in this plot was then used for the zfCD4-1 anti rabbit polyclonal antibody for further experiments.
