# Supplementary material for: First Demonstration of Antigen Induced Cytokine Expression by CD4-1+ Lymphocytes in a Poikilotherm: Studies in Zebrafish (Danio rerio)
Source: PLoS One. 2015 Jun 17;10(6):e0126378. doi: 10.1371/journal.pone.0126378 (PMC4470515; doi:10.1371/journal.pone.0126378)
Supplement: S1 Table — (PDF) [file pone.0126378.s007.pdf]

Table S1. Primers used in cDNA synthesis and RACE PCR for zfCD4 genes.

Primer name	Sequence (5’-3’)	Used for
Oligo-dT	TTTTTTTTTTTTTTTTVN	cDNA synthesis
Adapter-dT	CTCGAGATCGATGCGGCCGCTTTTTTTTTTTTTTTTVN	RACE PCR
Adapter	CTCGAGATCGATGCGGCCGC	
Oligo-dG	GGGGGGIGGGIIGGGIIG	
CD4-2.1raceF1	GTAGGAGCCTTTTCTGTGGTTC	
CD4-2.1raceF2	CAGGTACTGCAAATCCTCTTCC	
CD4-2.1raceR1	GCGTGTATTGTTTCCCTGACTG	
CD4-2.1raceR2	TGGGAATGTTTAGATGTCTCC	
CD4-2.2raceF1	GAAGGAGACCCAAACACTGAAG	
CD4-2.2raceF2	CAAGTGATGAAGGCAAGTGGAC	
CD4-2.2raceR1	CTTCGTCTGCTGTGTTCTTCAG	
CD4-2.2raceR2	AAGGTGGTCTGCTTTCCATTC	
CD4-1raceF1	GCTGACACGACTCTGAAGACAAG	
CD4-1raceF2	CTTGTCTTCAGAGTCGTGTCAGC	
CD4-1raceR1	CGCCAAATGACAAAATCAGAC	
CD4-1raceR2	TGTTTTCTCTGGGTAGGGTGAC	
