# Supplementary material for: First Demonstration of Antigen Induced Cytokine Expression by CD4-1+ Lymphocytes in a Poikilotherm: Studies in Zebrafish (Danio rerio)
Source: PLoS One. 2015 Jun 17;10(6):e0126378. doi: 10.1371/journal.pone.0126378 (PMC4470515; doi:10.1371/journal.pone.0126378)
Supplement: S2 Table — (PDF) [file pone.0126378.s008.pdf]

Table S2 . Primers used in real time PCR for gene expression in zebrafish.

Primer name	Sequence (5'-3')	Used for
EF-1α-F	CTGGAGGCCAGCTCAAACAT	Conventional PCR and Real Time PCR
EF-1α-R	ATCAAGAAGAGTAGTAGTACCG	
CD4-2.1-F	CTTCAATGTCACTCTGATAAATGTGC	
CD4-2.1-R	TTACGTGCCGTGTAGATCTG	
CD4-2.2-F	TTTGGAGTTGAACTTTGGATCTGGA	
CD4-2.2-R	GATCTCAGCTTTCTCACTCTCCTC	
CD4-1-F	CTGTTTCTGTTATAGACCTTGCC	
CD4-1-R	CTGTTTCTGTTATAGACCTTGCC	
Tbet-F	TCACCAACCATACCTCTC	
Tbet-R	GTATTCGGTCCCGTAAGC	
TcRα-F	TTACTGCGAGGAGACAGGC	
TcRα-R	TCCTCAGCCAGAAGATGCC	
MHC class Ⅱ β chain –F	CTGGAGTGGACACATTCTGCA	
MHC class Ⅱ β chain –R	CTCTTTACCATCTCTCAGCCAGG	
IgM-F	AGATCCAATACAAAGATACTATGC	
IgM-R	TGGTGAAATGGAATTGTGG	
CD8α-F	GGAGTACCAGGTGGGCTTTT	
CD8α-R	GAGGAAAAGTCCACAACCTC	
MCSF-R-F	CCAAAGCTCTCTTCTAAACTCACAC	
MCSF-R-R	GTACCTGTCGTTATGGTTGAAAAAC	
IFN-γ-F	GCATCGAAGAGCTCAAAGCTTAC	
IFN-γ-R	TCTGCTCACTTTCCTCAAGATTC	
GATA3-F	GAGCTGGGACCTCATGCGCAAAC	
GATA3-R	GATGCCCTCCTTCTTCATGGTG	
IL-4/13A-F	GAAGTGTGAGCATGATTATTTC	
IL-4/13A-R	CTCGTCTTGGTGGTTGTAAG	
IL-4/13B-F	CATCCAGAGTGTGAATGGGA	
IL-4/13B-R	TTCCAGTCCCGGTATATGCT	
IL-17A/F1-F	ACCTCCGCTTTCTTATGGTGAG	
IL-17A/F1-R	ATCTTACAGAAGCCCCCTCAG	
IL-17A/F2-F	GGAACTGGATACCGAAGTTTTC	
IL-17A/F2-R	CCTGTTTCAGCACCAGTATGTCC	
IL-22-F	CTACCTGCGATATGAAGTGC	
IL-22-R	CATTTCGTGCTGTATCAGTC	
