# Supplementary material for: Correction: First Demonstration of Antigen Induced Cytokine Expression by CD4-1+ Lymphocytes in a Poikilotherm: Studies in Zebrafish (Danio rerio)
Source: PLoS One. 2016 Dec 21;11(12):e0169149. doi: 10.1371/journal.pone.0169149 (PMC5176319; doi:10.1371/journal.pone.0169149)
Supplement: S2 Table — (DOCX) [file pone.0169149.s001.docx]

Table S2 . Primers used in real time PCR for gene expression in zebrafish.

| Primer name | Sequence (5’-3’) | Used for |
| --- | --- | --- |
| EF-1α-F | CTGGAGGCCAGCTCAAACAT | Conventional PCR  and  Real Time PCR |
| EF-1α-R | ATCAAGAAGAGTAGTAGTACCG |  |
| CD4-2.1-F | CTTCAATGTCACTCTGATAAATGTGC |  |
| CD4-2.1-R | TTACGTGCCGTGTAGATCTG |  |
| CD4-2.2-F | TTTGGAGTTGAACTTTGGATCTGGA |  |
| CD4-2.2-R | GATCTCAGCTTTCTCACTCTCCTC |  |
| CD4-1-F | CTGTTTCTGTTATAGACCTTGCC |  |
| CD4-1-R | CTGGTCGGTCTTAAATGAAACT |  |
| Tbet-F | TCACCAACCATACCTCTC |  |
| Tbet-R | GTATTCGGTCCCGTAAGC |  |
| TcRα-F | TTACTGCGAGGAGACAGGC |  |
| TcRα-R | TCCTCAGCCAGAAGATGCC |  |
| MHC class Ⅱ β chain –F | CTGGAGTGGACACATTCTGCA |  |
| MHC class Ⅱ β chain –R | CTCTTTACCATCTCTCAGCCAGG |  |
| IgM-F | AGATCCAATACAAAGATACTATGC |  |
| IgM-R | TGGTGAAATGGAATTGTGG |  |
| CD8α-F | GGAGTACCAGGTGGGCTTTT |  |
| CD8α-R | GAGGAAAAGTCCACAACCTC |  |
| MCSF-R-F | CCAAAGCTCTCTTCTAAACTCACAC |  |
| MCSF-R-R | GTACCTGTCGTTATGGTTGAAAAAC |  |
| IFN-γ-F | GCATCGAAGAGCTCAAAGCTTAC |  |
| IFN-γ-R | TCTGCTCACTTTCCTCAAGATTC |  |
| GATA3-F | GAGCTGGGACCTCATGCGCAAAC |  |
| GATA3-R | GATGCCCTCCTTCTTCATGGTG |  |
| IL-4/13A-F | GAAGTGTGAGCATGATTATTTC |  |
| IL-4/13A-R | CTCGTCTTGGTGGTTGTAAG |  |
| IL-4/13B-F | CATCCAGAGTGTGAATGGGA |  |
| IL-4/13B-R | TTCCAGTCCCGGTATATGCT |  |
| IL-17A/F1-F | ACCTCCGCTTTCTTATGGTGAG |  |
| IL-17A/F1-R | ATCTTACAGAAGCCCCCTCAG |  |
| IL-17A/F2-F | GGAACTGGATACCGAAGTTTTC |  |
| IL-17A/F2-R | CCTGTTTCAGCACCAGTATGTCC |  |
| IL-22-F | CTACCTGCGATATGAAGTGC |  |
| IL-22-R | CATTTCGTGCTGTATCAGTC |  |
